# Adiponectin Stimulates Exosome Release to Enhance Mesenchymal Stem-Cell-Driven Therapy of Heart Failure in Mice

**DOI:** 10.1016/j.ymthe.2020.06.026

**Published:** 2020-07-10

**Authors:** Yuto Nakamura, Shunbun Kita, Yoshimitsu Tanaka, Shiro Fukuda, Yoshinari Obata, Tomonori Okita, Hiroyuki Nishida, Yuki Takahashi, Yusuke Kawachi, Yuri Tsugawa-Shimizu, Yuya Fujishima, Hitoshi Nishizawa, Yoshinobu Takakura, Shigeru Miyagawa, Yoshiki Sawa, Norikazu Maeda, Iichiro Shimomura

**Affiliations:** 1 Department of Metabolic Medicine, Graduate School of Medicine, Osaka University, Osaka, Japan; 2 Department of Adipose Management, Graduate School of Medicine, Osaka University, Osaka, Japan; 3 ROHTO Pharmaceutical Co., Ltd. Osaka, Japan; 4 Department of Advanced Stem Cell Therapy, Graduate School of Medicine, Osaka University, Osaka, Japan; 5 Department of Biopharmaceutics and Drug Metabolism, Graduate School of Pharmaceutical Sciences, Kyoto University, Kyoto, Japan; 6 Department of Cardiovascular Surgery, Graduate School of Medicine, Osaka University, Osaka, Japan; 7 Medical Center for Translational Research, Osaka University Hospital, Osaka, Japan; 8 Department of Metabolism and Atherosclerosis, Graduate School of Medicine, Osaka University, Osaka, Japan

**Keywords:** adiponectin, exosome, extracellular vesicle, mesenchymal stem/stromal cell, MSC, heart failure, TAC model, T-cadherin

## Abstract

Mesenchymal stem/stromal cells (MSCs) are cultured adult stem cells that originally reside in virtually all tissues, and the gain of MSCs by transplantation has become the leading form of cell therapy in various diseases. However, there is limited knowledge on the alteration of its efficacy by factors in recipients. Here, we report that the cardioprotective properties of intravenously injected MSCs in a mouse model of pressure-overload heart failure largely depend on circulating adiponectin, an adipocyte-secreted factor. The injected MSCs exert their function through exosomes, extracellular vesicles of endosome origin. Adiponectin stimulated exosome biogenesis and secretion through binding to T-cadherin, a unique glycosylphosphatidylinositol-anchored cadherin, on MSCs. A pharmacological or adenovirus-mediated genetic increase in plasma adiponectin enhanced the therapeutic efficacy of MSCs. Our findings provide novel insights into the importance of adiponectin in mesenchymal-progenitor-mediated organ protections.

## Graphical Abstract

**Figure:**
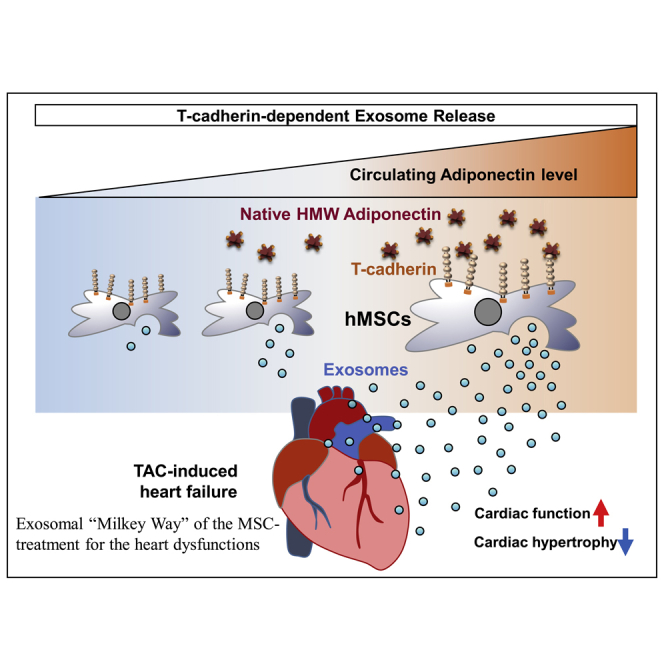

## Teaser

Mesenchymal stem/stromal cell (MSC) therapies are emergently under development for various diseases such as heart failure and also acute respiratory distress syndrome (ARDS) evoked by COVID-19 infections. Nakamura et al. uncovered the importance of adiponectin in the circulation and T-cadherin in human adipose-derived MSCs to enhance exosome synthesis reading to treat a heart failure model.

## Introduction

Adiponectin is an atypical factor that is present abundantly in the peripheral circulation and is exclusively secreted by adipocytes as a trimer, hexamer, and high-molecular-weight (HMW) multimer. Adiponectin circulates at the highest plasma concentrations known among adipocytokines/adipokines.^1^^,^^2^ Cross-sectional research in humans has demonstrated an inverse correlation between plasma adiponectin concentrations and body weight or body mass index (BMI).^3^^,^^4^ Functionally, adiponectin had been thought to play various metabolically important roles via adipoRs^2^ and dead cell opsonization through calreticulin.^5^ Clinical analyses have confirmed that HMW multimer (≥6 mers) adiponectin is the active form and that it possesses pleiotropic effects.^6^, ^7^, ^8^, ^9^ HMW multimer adiponectin is abundantly present in various tissues such as the heart, vascular endothelium, and skeletal muscles by binding with T-cadherin,^10^^,^^11^ a unique glycosylphosphatidylinositol (GPI)-anchored cadherin. We recently reported that native adiponectin binds to the cells expressing T-cadherin but not other proposed binding partners of adiponectin, such as adipoRs and calreticulin.^12^ The presence of HMW adiponectin (HMW-APN) in large amounts in these tissues is revealed as essential for adiponectin-mediated cardiovascular protection^13^, ^14^, ^15^ and skeletal muscle regeneration.^16^

Mesenchymal stem/stromal cells (MSCs) are cultured progenitor cells that originally reside in virtually all tissues and are thought to play a role in tissue homeostasis.^17^^,^^18^ A gain in MSCs is beneficial in a variety of diseases, including graft-versus-host disease,^19^ heart disease,^20^ type 1 diabetes,^21^ and type 2 diabetes.^22^ According to the public clinical trials database,^23^ more than 893 clinical trials have, so far, used MSCs in the treatment of diverse diseases. However, there is limited knowledge on the alteration of its efficacy by factors in recipients.

Exosomes are secreted small vesicles (50–150 nm) delimited by a lipid bi-layer generated by inward budding of the limiting membrane during endosome maturation into multivesicular bodies (MVBs) in the endocytic pathway. The exosomal release serves as a disposal pathway alternative to the lysosome.^24^ In addition to this, exosomes are thought to functionally mediate cell-to-cell communication under normal and pathological conditions by transferring active proteins, lipids, mRNAs, and small non-coding RNAs stably in various biofluids^25^^,^^26^ and play important roles in metabolic regulation pathways.^27^ Especially stem-cell-secreted exosomes are considered to be organ protective by modulating the immune function of the recipient cells^28^ or by stimulating the repair of recipient cells.^29^, ^30^, ^31^ A recent proteomics study indicated that T-cadherin is one of the most abundantly expressed proteins on the cell surface of MSCs.^32^

In our previous studies, we developed a new one-step purification method of the clinically important multimer adiponectin from serum^11^ and demonstrated that the use of adiponectin obtained through this procedure enhances exosome biogenesis and secretion.^27^^,^^33^ Furthermore, the systemic levels of exosome in the peripheral blood decreased by genetic loss of adiponectin and increased by overexpression of adiponectin *in vivo*.^27^^,^^33^ Adiponectin increased exosome biogenesis. It required the presence of T-cadherin but not AdipoRs.^16^^,^^27^^,^^33^ Adiponectin-induced stimulation of exosomes was also observed in muscle cells and correlated with improved muscle regeneration by adiponectin.^16^^,^^27^

The present study was designed to determine the role of adiponectin in the beneficial effects of human adipose tissue-derived mesenchymal stem cells (hMSCs) in heart failure. Systemic injection of hMSCs in the load-induced ventricular hypertrophy mouse model improved left ventricular cardiac function. This therapeutic effect of hMSCs largely depended on circulating adiponectin in recipient mice, T-cadherin expression in hMSCs, and ESCRT (endosomal sorting complex required for transport)-mediated exosome production by hMSCs. The use of peroxisome-proliferator-activated receptor γ (PPARγ) agonist or adenoviral overexpression of adiponectin increased plasma adiponectin levels and thereby enhanced MSC-induced cardioprotection, whereas no such protection was noted in adiponectin knockout (AKO) mice.

## Results

### Adiponectin Stimulated Exosome Production by hMSCs through T-cadherin

**Figure 1:**
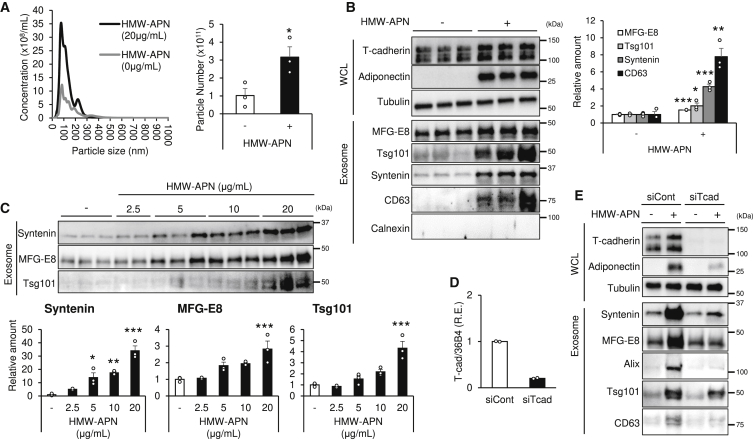
Adiponectin Stimulates Exosome Production by hMSCs through T-cadherin Exosomes were isolated from the culture medium of hMSCs as described in Materials and Methods. Exosome and whole-cell lysate (WCL) were lysed after 48 h with or without high-molecular-weight adiponectin (HMW-APN, 20 μg/mL). (A) NTA (n = 3). (B) Western blot analysis with the indicated antibodies of typical exosome markers, adiponectin (APN), and T-cadherin (T-cad), as indicated (n = 3). (C) hMSCs were treated with HMW-APN for 48 h in a dose-dependent manner, and the isolated exosomes were subjected to western blot analysis using the indicated antibodies (n = 2–3). (D) qPCR analysis of control and T-cad (CDH13) RNAi-transfected hMSCs at 48 h after transfection (n = 2). (E) Exosomes and WCL were collected from T-cad RNAi-transfected cells with or without APN. The proteins were subjected to western blot analysis with the indicated antibodies. Representative immunoblots are shown. Data are mean ± SEM. The results of the experiment were tested in two separate trials. For (A) and (B), Student’s t test; for (C), one-way analysis of variance with Dunnett’s multiple comparisons. ∗p < 0.05; ∗∗p < 0.01; ∗∗∗p < 0.001.

In our previous study, we revealed that adiponectin enhances the exosome biogenesis accompanying an increase in the particle number of exosomes.^33^ First, we measured the particle number of exosomes produced from hMSCs by nanoparticle tracking analysis (NTA). HMW-APN increased the exosome-sized particle production from hMSCs *in vitro* (Figure 1A). We examined T-cadherin protein expression and accumulation of adiponectin in hMSCs (Figure 1B, WCL [whole-cell lysate]). Western blot analysis confirmed that hMSCs express T-cadherin, as reported previously,^32^ and accumulate HMW-APN purified from mouse serum following 48-h incubation (Figure 1B, WCL).^11^ Next, we tested whether adiponectin enhanced exosome production from hMSCs (Figure 1B, Exosome). HMW-APN markedly increased exosome production, judging from the levels of classic exosome markers, such as syntenin, milk fat globule-epidermal growth factor 8 (MFG-E8), CD63, and tumor susceptibility gene 101 (Tsg101) in exosome fractions obtained from the conditioned medium of hMSCs (Figure 1B, Exosome), which is similar to the results we previously reported in cultured endothelial^33^ and muscle cells.^16^ In this condition, calnexin, an endoplasmic reticulum protein, was not detected in the exosome fraction, indicating successful exosome isolation (Figure 1B, Exosome). The HMW-APN-mediated exosome production was dose dependent within the range of physiological plasma HMW-APN concentrations in control subjects (Figure 1C). Similar to the results reported in endothelial^33^ and muscle^16^ cells, the accumulation of HMW-APN was significantly reduced by T-cadherin knockdown (Figures 1D and 1E), which was accompanied by a reduction of HMW-APN-mediated exosome production (Figure 1E).

### Increase of Circulating Exosomes by Intravenous hMSC Injection Accumulating in Lung

**Figure 2:**
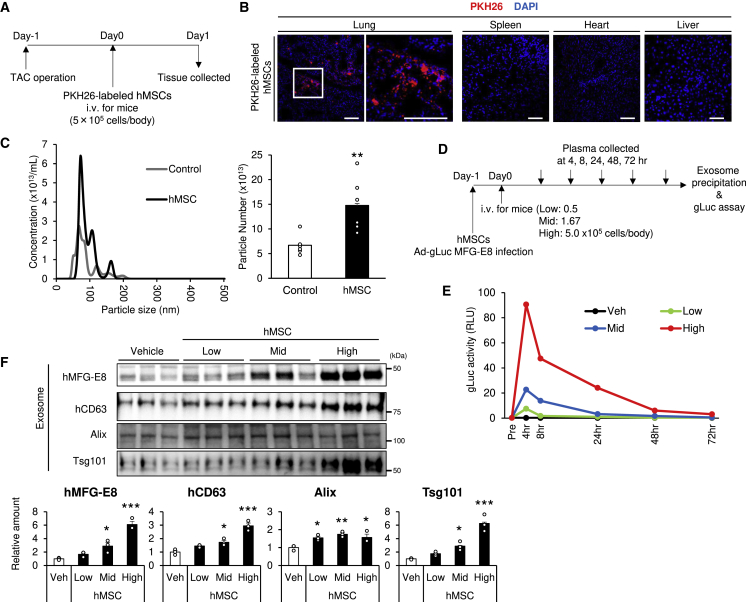
Increase of Circulating Exosomes by Intravenous hMSC Injection Accumulating in the Lung (A) Experimental design for PKH26-labeled hMSC injection into the transverse aortic constriction (TAC) model. PKH26-labeled hMSCs were injected at a concentration of 5.0 × 10^5^ cells per body via the tail vein. (B) Representative images of frozen sections injected with PKH26-labeled hMSCs (scale bars, 100 μm). (C) The serum was collected at 4 h after hMSC injection via the tail vein (5.0 × 10^5^ cells per body). Exosome was prepared from the serum and analyzed by NTA (n = 6). (D) Experimental design: adenovirus Gaussia luciferase (Ad-gLuc)-MFG-E8 infected cells were injected into wild-type (WT) mice. The transfected hMSCs were injected at concentrations of 0.5 × 10^5^ (low), 1.67 × 10^5^ (mid), and 5.0 × 10^5^ (high) cells per body. Blood samples were collected at 4, 8, 24, 48, and 72 h, and plasma was extracted. Plasma exosomes were precipitated using Exo Quick and ultracentrifugation as described in Materials and Methods. (E) gLuc activity of plasma exosome was analyzed using luminometer (pooled sample of four WT mice). Veh, vehicle. (F) Mice were injected with hMSCs via the tail vein, and blood was collected at 4 h after transplantation. Serum exosome was precipitated by Exo Quick and ultracentrifugation, as described in Materials and Methods. Serum exosomes were subjected to western blot analysis using the indicated antibodies of exosome markers (n = 3). Data are presented as mean ± SEM. The results of the experiment were tested in two separate trials. For (C), Student’s t test; for (F), one-way analysis of variance with Dunnett’s multiple comparisons. ∗p < 0.05; ∗∗p < 0.01; ∗∗∗p < 0.001.

Previous studies have demonstrated that the vast majority of intravenously (i.v.) injected MSCs accumulate in the lung microvasculature;^34^^,^^35^ hence, the therapeutic effects of hMSCs in this study are supposed to rely on secreted factors.^36^^,^^37^ To clarify the localization of injected hMSCs, we i.v. injected PKH26-labeled hMSCs for transverse aortic constriction (TAC) in mice and evaluated the sections in the lung, spleen, heart, and liver (Figures 2A and 2B). In accordance with the previous studies,^38^ injected hMSCs were densely localized to the lung, although such dense images were not observed in other organs, like the spleen, heart, and liver (Figure 2B).

When we checked the number of circulating exosomes by NTA, the injection of hMSCs significantly increased the exosome-sized (50–150 nm) particle numbers in circulation at 4 h after systemic hMSC injection (Figure 2C). To determine the amount of exosome specifically produced by hMSCs transplanted *in vivo*, we developed a new method to detect hMSC-derived exosomes specifically from blood circulation in mice. Gaussia luciferase fused with MFG-E8 (gLuc-MFG-E8) was successfully used to label exosomes *in vitro* and to monitor labeled exosomes in the circulation using small volumes of plasma.^39^^,^^40^ We exploited this technique to label exosomes produced by the transplanted hMSCs by introducing gLuc-MFG-E8 cDNA into hMSCs (Figures 2D and 2E). Exosome-associated gLuc activity was successfully monitored during the time course study following intravenous injection of gLuc-MFG-E8-expressing hMSCs (Figure 2E). The activity increased with injection of higher cell numbers and gradually decreased within 3 days (Figure 2G). Since exosome-associated gLuc activity in adenovirus-infected cells was in parallel with the growth of hMSCs during 72-h culture after infection *in vitro* (Figure S1), it is likely that the observed *in vivo* change in the exosomes is not due to loss of gLuc-MFG-E8 gene expression in hMSCs but rather to the decrease in viability or exosome production by hMSCs. In addition, the increase of circulating exosomes by the hMSCs was also detected by western blot analysis of exosome fractions obtained by ultracentrifugation using exosome marker antibodies (Figure 2G). Exosome markers, such as ALG-2 interacting protein (Alix) and Tsg101 (antibodies of these can detect both human and mouse proteins), also significantly increased following hMSC injection (Figure 2G). These findings suggest that not only hMSC-derived exosomes but also whole amounts of exosome in blood increased by hMSC injection.

### Injection of hMSCs Ameliorated Cardiac Dysfunction in Pressure-Overload-Induced Heart Failure

**Figure 3:**
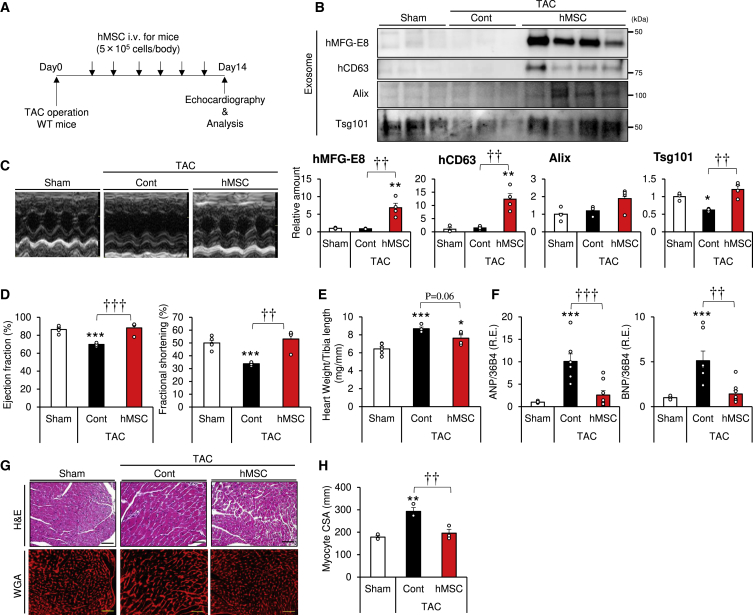
Intravenous Injection of hMSCs Improves Cardiac Function in Mice with Pressure-Overload-Induced Heart Failure (A) Experimental design for hMSC delivery into the transverse aortic constriction (TAC) model. hMSCs were injected at a concentration of 5.0 × 10^5^ cells per body via the tail vein. The injection was repeated 6 times at 2- to 3-day intervals within a period of 2 weeks. Echocardiography was performed at day 14. (B) Serum exosomes were subjected to western blot analysis with the indicated antibodies of exosome markers (n = 3–4). (C–H) Comparison of heart tissue of WT mice with or without injection of hMSCs at 2 weeks after TAC or sham surgery. (C) Representative images of echocardiography. (D) Ejection fraction (EF) and fractional shortening (FS) measured at 2 weeks after TAC or sham surgery (n = 4–6). (E) Heart weight per tibia length ratio (n = 4–6). (F) Relative expression of heart failure markers of the indicated mice (n = 7–8). (G) Representative images of hematoxylin and eosin (H&E)-stained and wheat germ agglutinin (WGA)-stained sections of the apical heart (scale bars, 50 mm). (H) Myocyte cross-sectional area (CSA) calculated by BZ-X analyzer software (n = 3). Data are mean ± SEM. All experiments in this figure were tested in two separate trials. ∗p < 0.05; ∗∗p < 0.01; ∗∗∗p < 0.001 versus sham. ^††^p < 0.01; ^†††^p < 0.001 between groups, by one-way analysis of variance with post hoc Tukey’s multiple comparisons.

Next, we evaluated the therapeutic effect of hMSCs in a pressure-overload-induced heart failure animal model. The model was prepared by TAC, a procedure known to induce chronic heart failure. To evaluate the therapeutic efficacy of hMSCs, we injected hMSCs via the lateral tail vein within a period of 2 weeks at 2- to 3-day intervals (Figure 3A). Cell dose and intervals were determined according to the disappearance of hMSC-produced exosomes (Figures 2E and 2F), as well as a pilot dose-finding study (Figure S2). Serum exosomes fractionated by ultracentrifugation were subjected to western blotting. Interestingly, Tsg101 was markedly decreased following TAC (Figure 3B). Injection of hMSCs significantly increased the amounts of exosome markers (Figure 3B), and such effects appeared to be hMSC cell number dependent (Figure S2).

We evaluated cardiac function in the aforementioned mice by ultrasonic echocardiography (Figure 3C). TAC resulted in a significant decrease in cardiac function, including ejection fraction (EF) and fractional shortening (FS) (Figure 3D), and i.v. injected hMSCs improved these cardiac functions (Figure 3D). TAC also resulted in a significant increase in heart weight, compared to sham operation, and injection of hMSCs tended to decrease the heart-weight/tibia-length ratio (Figure 3E). On a molecular level, TAC significantly increased the expression levels of heart failure markers (ANP and BNP), compared to sham operation (Figure 3F), and injection of hMSCs significantly decreased these markers. We also measured the cross-sectional area (CSA) by wheat germ agglutinin (WGA) plasma membrane staining to evaluate cardiomyocyte hypertrophy (Figure 3G). The results demonstrated significant TAC-associated left ventricular hypertrophy, while the injection of hMSCs prevented this effect (Figures 3G and 3H). Moreover, hMSC treatment during the first 2 weeks improved cardiac function even at 4 weeks (Figure S3). Taken together, these results indicate that injection of hMSCs improves left ventricular hypertrophy in the chronic heart failure mouse model, which is associated with an increase of exosomes in the circulation.

### Dependence of hMSC-Related Cardioprotective Effect on Circulating Adiponectin

**Figure 4:**
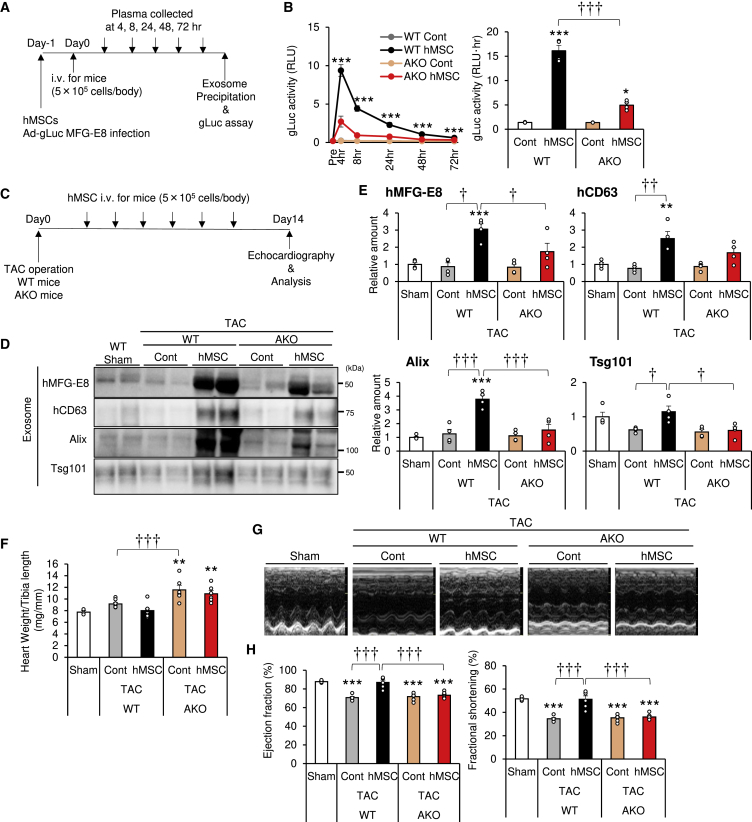
Importance of Circulating Adiponectin for the Cardioprotective Effects of hMSCs (A) Experimental design for adenovirus Gaussia luciferase (Ad-gLuc)-MFG-E8-infected hMSCs. Infected hMSCs were injected i.v. at 5.0 × 10^5^ cells per body into adiponectin knockout (AKO) and wild-type (WT) mice via the tail vein. Blood was collected at 4, 8, 24, 48, and 72 h. Plasma exosomes were precipitated by Exo Quick and ultracentrifugation, as described in Materials and Methods. (B) gLuc activity of plasma exosome was analyzed using a luminometer. The area under the curve (AUC) was calculated (n = 3–4). (C–H) hMSCs were injected i.v. at a concentration of 5.0 × 10^5^ cells per body via the tail vein in the transverse aortic constriction (TAC) model in AKO and WT mice. (C) Experimental design for hMSC delivery into the TAC model. The injection was repeated six times at 2- to 3-day intervals within a period of 2 weeks. Echocardiography was performed at day 14. (D) Serum exosomes were subjected to western blot analysis with the indicated antibodies of exosome markers. (E) Exosome marker levels from western blots (n = 3–4). (F) Heart weight per tibia length ratio (n = 3–10). (G) Representative images of echocardiography. (H) Ejection fraction (EF) and fractional shortening (FS) estimated at 2 weeks after TAC or sham surgery (n = 3–6). Data are mean ± SEM. The experiments were tested in two separate trials. ∗p < 0.05; ∗∗p < 0.01; ∗∗∗p < 0.001 versus sham. ^†^p < 0.05; ^††^p < 0.01; ^†††^p < 0.001 between groups, by one-way analysis of variance with post hoc Tukey’s multiple comparisons.

First, we evaluated the effect of circulating adiponectin on exosome production by hMSCs *in vivo*. Adenovirus-gLuc-MFG-E8-infected hMSCs were injected i.v. in wild-type (WT) and AKO mice under normal conditions using the same protocol described in Figure 2E (Figure 4A). Exosome-associated gLuc activity was significantly lower in AKO mice than in WT mice at all time points (up to 72 h) (Figure 4B). The area under the curve (AUC) of gLuc activity was significantly higher after hMSC injection in WT mice and significantly higher than in AKO mice (Figure 4B).

Second, to determine the role of adiponectin in the therapeutic effects of hMSCs, hMSCs were injected into AKO mice with load-induced cardiac hypertrophy using the same protocol described in Figure 3A (Figure 4C). hMSCs significantly increased all exosome markers tested in WT mice, but such increase was significantly attenuated in AKO mice (Figures 4D and 4E). These results highlight the importance of circulating adiponectin in normal exosome production from hMSCs. TAC was associated with the worsening of cardiac hypertrophy in AKO compared with WT mice, confirming the findings of a previous study (Figure 4F).^13^ We also evaluated cardiac function by ultrasonic echocardiography (Figures 4G and 4H). At postoperative day 14, cardiac function was equally decreased by TAC in both WT and AKO mice (Figures 4G and 4H), although a previous study reported that further prolongation of pressure overload caused more severe cardiac dysfunction in AKO than WT mice.^13^ Injection of hMSCs improved cardiac function in WT mice but not in AKO mice (Figures 4G and 4H; Table S1). These results indicate that hMSCs require circulating adiponectin to produce many exosomes and effectively improve cardiac functions in mice.

### High Adiponectin Levels Augmented the Cardioprotective Effect of hMSCs

**Figure 5:**
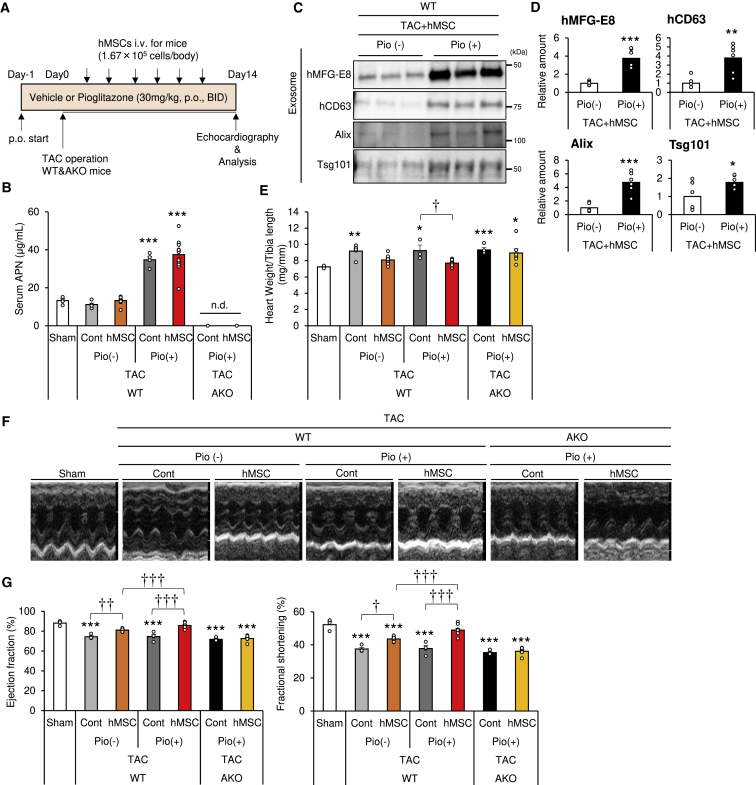
High Adiponectin Levels Augment the Cardioprotective Effects of hMSCs (A) Experimental design for hMSC intravenous injection and simultaneous oral (p.o.) administration of pioglitazone (Pio; 30 mg/mL, BID) into mice of the transverse aortic constriction (TAC) model. hMSCs were injected at a concentration of 1.67 × 10^5^ cells per body via the tail vein. The injection was repeated 6 times at 2- to 3-day intervals within a period of 2 weeks. Echocardiography was performed at day 14. (B) Plasma adiponectin levels were analyzed by ELISA (n = 4–13). n.d., not detected. (C) Representative immunoblots. Serum exosomes from WT mice were subjected to western blot analysis with the indicated antibodies of exosome markers. (D) Exosome marker levels from western blots (n = 6). (E) Heart weight per tibia length ratio (n = 4–13). (F) Representative images of echocardiography. (G) Ejection fraction (EF) and fractional shortening (FS) at 2 weeks after TAC or sham surgery (n = 3–9). Data are mean ± SEM. ∗p < 0.05; ∗∗p < 0.01; ∗∗∗p < 0.001 versus sham. ^†^p < 0.05; ^††^p < 0.01; ^†††^p < 0.001 between groups, by one-way analysis of variance with post hoc Tukey’s multiple comparisons.

The therapeutic effects of injected hMSCs are mediated by circulating adiponectin. Importantly, exosome production increased in a dose-dependent manner by purified HMW-APN *in vitro* (Figure 1C). Next, we examined whether exogenous adiponectin enhances the therapeutic efficacy of injected hMSCs. To test this, mice were orally administrated pioglitazone, a well-known thiazolidinedione class of PPARγ agonists, from 1 day before TAC to 2 weeks after TAC (Figure 5A). To evaluate the improvement in the therapeutic effect of hMSCs, cells were injected at a submaximal number of 1.67 × 10^5^ cells per body (one third of that used in the other studies) within a period of 2 weeks at 2- to 3-day intervals (Figure 5A). Two weeks after administration of pioglitazone, serum adiponectin levels were significantly higher in the pioglitazone-treated group compared with the vehicle group (vehicle: 12.7 ± 0.5 μg/mL; pioglitazone: 36.8 ± 1.7 μg/mL). No such changes were noted in the AKO mice (Figure 5B). Pioglitazone, in combination with hMSC injection, significantly increased all exosome markers tested in the exosome fractions obtained from serum (Figures 5C and 5D). These results demonstrate that hMSCs plus pioglitazone treatment enhanced hMSC-based exosome production.

Heart weight was significantly larger in TAC-operated mice compared to sham-operated mice, regardless of pioglitazone administration (Figure 5E). Compared to the TAC-operated control mice, hMSC injection tended to decrease heart weight in the vehicle group (Figure 5E) and significantly decrease heart weight in the pioglitazone group (Figure 5E). TAC decreased cardiac function, as measured by EF and FS, regardless of pioglitazone administration (Figures 5F and 5G). Compared to these TAC-operated control mice, hMSCs with or without pioglitazone significantly improved cardiac function (Figures 5F and 5G). However, compared to the vehicle, hMSCs with pioglitazone significantly improved cardiac function (Figures 5F and 5G, orange versus red; Table S2). Importantly, all such experiments in AKO mice showed that pioglitazone had no effect on heart weight or cardiac function (Figures 5E–5G), strongly suggesting that pioglitazone indirectly affects the therapeutic effects of hMSCs through elevation of circulating adiponectin.

We reported previously that adenoviral adiponectin overexpression increased plasma exosome levels.^33^ We showed here that the adenoviral adiponectin overexpression, but not β-galactosidase, increased plasma adiponectin levels (Figures S4A and S4B) and significantly augmented the cardioprotective effects of MSCs (Figures S4C–S4E). Collectively, these results demonstrate that hyperadiponectinemia can improve the efficacy of injected MSCs through increased exosome production.

### T-cadherin Expression Is Important for hMSC/Adiponectin-Related Increase in Exosome Production *In Vivo*

**Figure 6:**
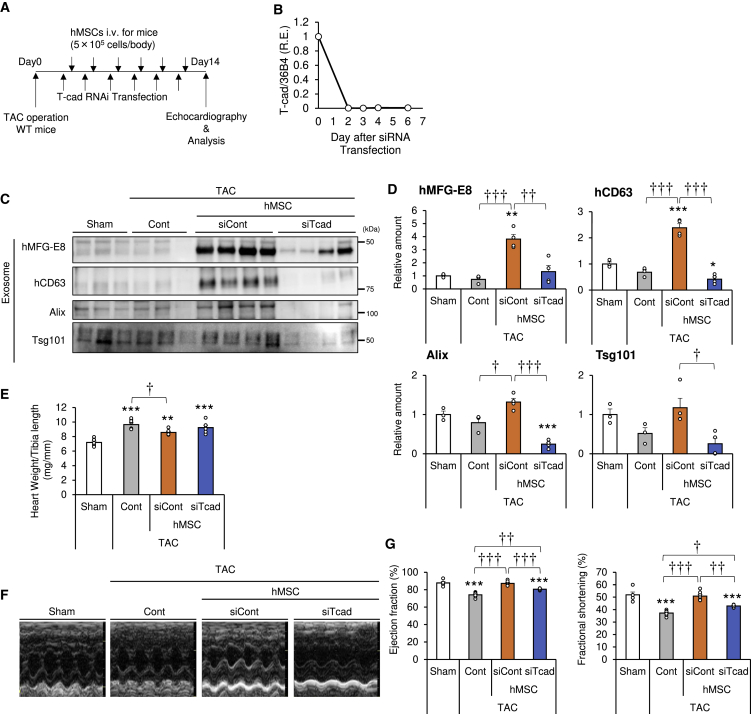
Importance of T-cadherin in hMSCs for Adiponectin-Induced Exosome Production and Cardioprotection *In Vivo* (A) Experimental design for intravenous injection of hMSCs in the transverse aortic constriction (TAC) model. hMSCs were transfected with control or T-cad (CDH13) RNAi and injected at a concentration of 5.0 × 10^5^ cells per body via the tail vein the day after transfection. Injections were repeated six times at 2- to 3-day intervals within a period of 2 weeks. Echocardiography was performed at day 14. (B) Relative expression of T-cadherin in hMSCs after RNAi transfection at an indicated time points (n = 2). (C) Representative immunoblots. Serum exosomes were subjected to western blot analysis with the indicated antibodies to exosome markers. (D) Serum exosome marker levels from western blots (n = 3–4). (E) Heart weight per tibia length ratio (n = 6–8). (F) Representative images of echocardiography. (G) Ejection fraction (EF) and fractional shortening (FS) at 2 weeks after TAC or sham surgery (n = 6–8). Data are mean ± SEM. All subjects in this figure were tested in two separate trials. ∗p < 0.05; ∗∗p < 0.01; ∗∗∗p < 0.001 versus sham. ^†^p < 0.05; ^††^p < 0.01; ^†††^p < 0.001 between groups, by one-way analysis of variance with post hoc Tukey’s multiple comparisons.

To investigate the role of T-cadherin on the effects of hMSCs, we injected T-cadherin knockdown hMSCs into WT mice with a load-induced ventricular hypertrophy model (Figure 6A). In this set of experiments, small interfering RNA (siRNA) transfection was used to markedly reduce T-cadherin expression and the knockdown efficacy was confirmed to persist for more than 6 days in a cell-culture study (Figure 6B). The use of the T-cadherin knockdown hMSCs produced fewer exosomes *in vitro* in response to adiponectin (Figure 1E). Control siRNA (siCont)-transfected or T-cadherin siRNA (siTcad)-transfected hMSCs were injected i.v. after TAC operation (Figure 6A), followed by measurement of serum exosome levels. siCont hMSC injection significantly increased exosome markers hMFG-E8, hCD63, and Alix and tended to increase Tsg101 (Figures 6C and 6D). In comparison, injection of T-cadherin knockdown hMSCs significantly decreased all those exosome markers (Figures 6C and 6D) and exosome-sized particle numbers in the blood to the level of the vehicle control (Figure S5). These results demonstrate the importance of T-cadherin expression on the exosome production function of the hMSCs *in vivo*. TAC, but not sham, operation was associated with a significant increase in heart weight, compared to sham-operated mice. Heart weight significantly decreased by siCont hMSC injection, but not by siTcad hMSC injection (Figure 6E).

Next, we examined cardiac function by echocardiography (Figures 6F and 6G). TAC significantly decreased cardiac function, such as EF and FS, while siCont hMSC injection improved cardiac function (Figure 6G), similar to non-transfected hMSCs (Figures 3D and 4H). In contrast, T-cadherin knockdown in hMSCs significantly attenuated such an effect of hMSCs (Figure 6G; Table S3), demonstrating that the observed cardioprotective effects of hMSCs are mediated through T-cadherin expression. Collectively, these results highlight the importance of T-cadherin expression on MSCs for both normal exosome production and the cardioprotective function.

### Exosomes Mediated the Cardioprotective Effects of hMSCs

**Figure 7:**
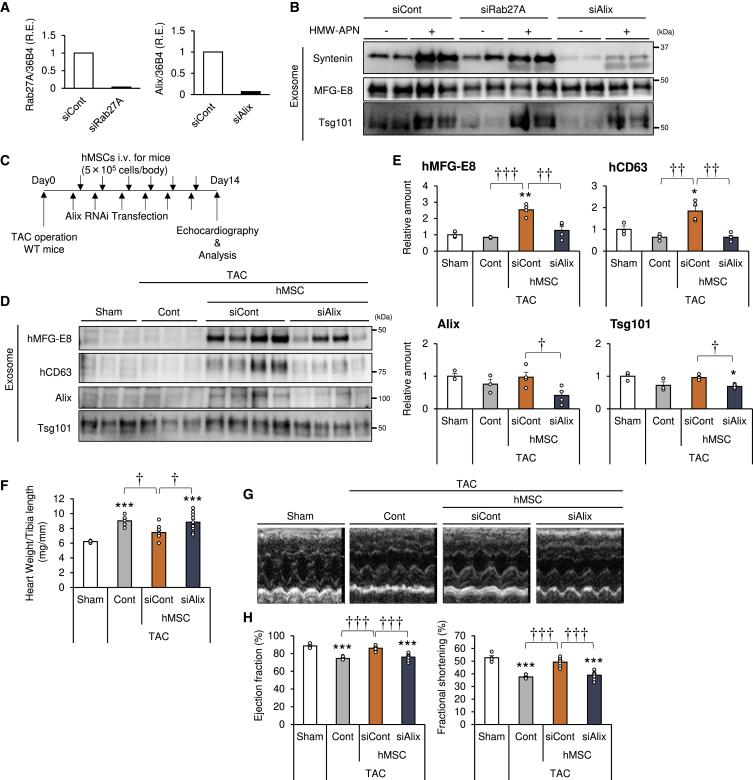
Exosomes Mediate the Cardioprotective Effects of hMSCs (A) hMSCs were transfected with RAB27A or Alix (PDCD6IP) RNAi and subjected to qPCR analysis at 4 days after RNAi transfection (n = 1). (B) Exosomes in culture media with or without HMW-APN (20 μg/mL) for 48 h were precipitated by ultracentrifugation and subjected to western blot analysis with the indicated antibodies against various exosome markers (n = 2). (C) Experimental design for intravenous injection of hMSCs in the transverse aortic constriction (TAC) model. hMSCs were transfected with Alix RNAi and injected at 5.0 × 10^5^ cells per body via the tail vein the day after transfection. Intravenous injection was repeated six times at 2- to 3-day intervals within a period of 2 weeks. Echocardiography was performed at day 14. (D) Representative immunoblots. Serum exosomes were subjected to western blot analysis with the indicated antibodies against exosome markers. (E) Serum exosome marker levels from western blots (n = 3–4). (F) Heart weight per tibia length ratio (n = 6–10). (G) Representative images of echocardiography. (H) Ejection fraction (EF) and fractional shortening (FS) at 2 weeks after TAC or sham surgery (n = 4–9). Data are mean ± SEM. *p < 0.05; **p < 0.01; ***p < 0.001 versus sham. ^†^p < 0.05; ^††^p < 0.01; ^†††^p < 0.001 between groups, by one-way analysis of variance with post hoc Tukey’s multiple comparisons.

The therapeutic effects of MSCs are considered to depend largely on their secretomes, such as cytokines and exosomes. Loss of adiponectin in the circulation or T-cadherin expression in hMSCs significantly attenuates the therapeutic effects of hMSCs as described earlier. To evaluate the importance of hMSCs-derived exosomes, we examined the effects of injection of exosome-deficient hMSCs on load-induced ventricular hypertrophy in mice. Various cellular machineries are known to play a role in exosome biogenesis and/or secretion, depending on the cell type. These include ESCRT components (e.g., Alix and syntenin), vesicle transport molecules (e.g., Rab27a and Rab27b), and ceramide metabolism proteins (e.g., neutral sphingomyelinase and sphingosine 1-phosphate receptor).^41^ Thus, we examined the effects of knockdown of several key pieces of the machinery of exosome biogenesis in hMSCs (Figure S6). The knockdown efficiency of each siRNA was confirmed by qPCR analysis. All of the aforementioned components, with the exception of neutral sphingomyelinase, suppressed the expression of the respective mRNA (Figures 7A and S6). Under such conditions, Alix knockdown decreased basal and adiponectin-stimulated exosome production (Figures 7B and S6) but did not alter cytokine secretion and multilineage differentiation potential (Figures S7 and S8). With regard to exosome production, Alix forms an important component of the ESCRT complex.^42^ TAC operation was followed by injection of siCont-transfected or Alix-siRNA-(siAlix)-transfected hMSCs (Figure 7C) and measurement of serum exosome level (Figures 7D and 7E). Injection of siCont hMSCs significantly increased exosome markers hMFG-E8 and hCD63 and tended to increase Alix and Tsg101. In contrast, the use of Alix-knockdown hMSCs significantly decreased all the aforementioned exosome markers (Figures 7D and 7E). Furthermore, heart weight increased significantly after TAC, compared to sham operation, and significantly decreased by injection of siCont hMSCs. On the other hand, the use of Alix-knockdown hMSCs significantly attenuated such effects (Figure 7F). We also used echocardiography to evaluate the cardioprotective effects of the exosome-deficient hMSCs (Figure 7G). As shown in Figures 3D, 4H, 5G, and 6G, TAC decreased cardiac function, as determined by EF and FS, and injection of siCont hMSCs significantly improved cardiac function (Figure 7H). In contrast, Alix-knockdown hMSCs significantly attenuated the cardioprotective effects of hMSCs (Figure 7H; Table S4). Collectively, these results indicate that hMSCs require exosome biogenesis for both normal exosome production and therapeutic effects in the heart failure model.

### MicroRNAs in Exosomes of hMSCs

**Figure 8:**
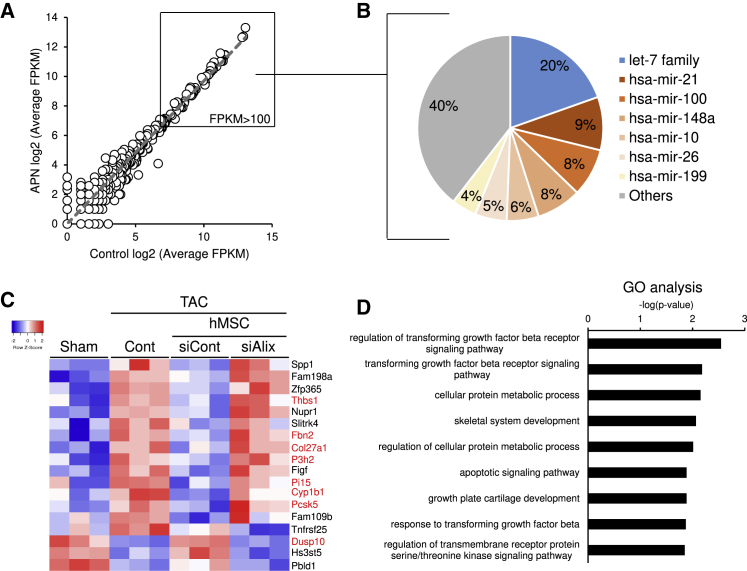
miRNAs in Exosomes from hMSCs (A) hMSC-derived exosomes were collected from the conditioned media after 48-h cultures. The most abundant miRNAs in hMSCs-derived exosomes. (B) Scatterplot showing the average fragments per kilobase of transcript per million fragments mapped (FPKM; log_2_ scale) of exosomes from the control hMSCs (abscissa) compared with exosomes from adiponectin-stimulated hMSCs (ordinate). (C) Expression profiles of the differential genes in the hearts of mice with the indicated groups under sham or TAC operation at 2 weeks. The color scale shows *Z*-scored FPKM, representing the mRNA level of each gene in blue (low)-white-red (high). The genes were expressed as fold change ≥ 1.5 and p < 0.05 between sham and TAC Cont, and between TAC Cont and TAC siCont hMSC groups. (D) Gene ontology (GO) analysis of RNA-seq transcriptome in the hearts of TAC and TAC+hMSC groups. Data are entries with −log (p value) > 2.5 and absolute *Z* score of ≥ 1.8.

MicroRNA (miRNA) mediates intercellular communication with exosomes.^25^^,^^26^ We performed miRNA sequencing (miRNA-seq) and compared the miRNA profile of hMSCs-produced exosomes in the presence or absence of 20 μg/mL adiponectin *in vitro* (Figure 8A). The resultant scatterplot suggested that adiponectin had little or no effect on the miRNA profile of the produced exosomes (Figure 8A). Among the meaningfully abundant miRNAs (fragments per kilobase of exon per million mapped fragments [FPKM] > 100), over half were accounted for by the seven highest expressing miRNAs and their families, including let-7 family, miR-21, -100, -148a, -10, -26, and -199 (Figure 8B). These miRNAs are known to mediate cardiovascular protection.^43^, ^44^, ^45^, ^46^

Next, we evaluated the change in cardiac transcriptome induced by hMSCs *in vivo*. Since TAC resulted in cardiac dysfunction, hMSCs with siCont significantly protected against such dysfunction, and Alix-knockdown hMSCs had poor effect on the heart, we extracted the differentially expressed genes evident on the heatmap that were associated with the aforementioned changes in cardiac function (Figure 8C). Ontology analysis of all genes in the heatmap indicated association with “TGFβ signaling,” “cellular metabolic process,” and “apoptotic signaling pathway” (Figure 8D). We also analyzed the predicted biological target of miRNAs by searching the TargetScan database. The results identified eight genes (Figure 8C, red) among the differentially expressed genes that were probable targets of miRNAs in hMSC-derived exosomes (Figure 8A, FPKM > 100; Table S5). Considered together, these results suggest that hMSCs-derived exosomes regulated, at least in part, the fibrogenic and adhesion pathways, and the cellular metabolic process, in the damaged heart.

## Discussion

Here, we report that adiponectin stimulates exosome release to enhance MSC-driven therapy of heart failure in mice. This therapeutic effect of hMSCs was dependent on circulating adiponectin in recipient mice, T-cadherin expression in hMSCs, and ESCRT-mediated exosome production by hMSCs and was associated with increases in hMSC-derived exosomes in the circulation. An increase in plasma adiponectin level by the administration of PPARγ agonist or adenoviral overexpression enhanced the therapeutic efficacy of hMSCs in the cardiac hypertrophy model. Our findings provide both a method to enhance MSC therapy and novel insights into the importance of exosome-mediated pleiotropic organ protection by adiponectin.

We demonstrated that plasma adiponectin level is a critical determinant of MSC-related effects in a heart failure model. Loss of adiponectin diminished, and its increase enhanced, the therapeutic efficacy of hMSCs. It will be intriguing to determine the baseline plasma adiponectin levels between responders and non-responders in hMSC-based clinical trials. Notably, the cardioprotective effects of hMSCs were not significant in adiponectin-deficient mice; hence, one can conclude that the PPARγ agonist used in this study did not directly affect hMSCs but exerted the observed cardioprotective effect by increasing plasma adiponectin levels. We reported previously that the use of a PPARγ agonist enhanced the therapeutic effect of a surgical fibrin graft containing stromal-vascular cells in a chronic myocardial infarction model.^47^ Another group reported that a PPARγ agonist enhanced the therapeutic effects of MSCs in the type 2 diabetes model through a decrease in plasma glucose levels.^48^ Importantly, the increase of adiponectin by pioglitazone treatment accompanied the improved cardiac function only when hMSCs were injected (Figures 5F and 5G). Further, in AKO mice, hMSC transplantation with a PPARγ agonist did not affect cardiac functions (Figures 5F and 5G). Taken together, a PPARγ-agonist-mediated adiponectin increment strengthened the efficacy of hMSC transplantation rather than a PPARγ agonist or adiponectin affected independently of transplanted hMSCs. Therefore, no equivalent MSC-like cells and other T-cadherin-expressing cells in mice appeared to respond to adiponectin in our TAC model, except by superphysiological overexpression of adiponectin by adenovirus (Figure S4). Our study in the normoglycemic heart failure model indicates that exosome stimulation through an increase in circulating adiponectin level should be one of the key mechanisms of such action of PPARγ agonists and provide the future opportunity of advanced therapeutic applications.

Although the initial applications selected for MSC therapy focused on their multi-lineage differentiation capacity, the importance of secreting mediators has been gained momentum recently.^49^ MSC therapy reduced the severity of inflammation, apoptosis, and fibrosis in numerous disease models, despite little differentiation and engraftment in the injured tissue.^50^ Thus, it has been hypothesized that regeneration must be due to the secretion of mediators rather than differentiation.^51^ Indeed, MSCs secrete a variety of bioactive molecules, such as cytokines, chemokines, growth factors, and exosomes.^52^ In our study, we used a systemic injection of hMSCs and confirmed that the vast majority of them accumulated in the lung.^34^^,^^35^ Hence, the effects of hMSCs are supposed to mainly rely on paracrine-secreting mediators. More specifically, genetic loss of Alix, an ESCRT machinery important for exosome biogenesis,^42^ resulted in markedly diminished exosome production by MSCs *in vivo* and therapeutic effects on the heart failure model, implicating the exosome in mediating MSC effects. Our loss-of-exosome study is the first direct demonstration that exosomes mediate the paracrine therapeutic activity of injected MSCs. Moreover, we confirmed that neither Alix knockdown nor adiponectin treatment altered cytokine secretion from cultured MSCs and multilineage differentiation potential (Figures S7 and S8).

Functionally, exosomes mediate cell-to-cell communication under normal and pathological conditions, by transferring several factors, like active proteins, lipids, and small non-coding RNAs, stably in various biofluids.^25^^,^^26^ The results of the miRNA-seq of hMSCs-derived exosomes indicate that these extracellular structures contain large amounts of the let-7 family and miR-21, -148, and -10 (Figure 8), which are known to be beneficial for cardiovascular disease.^44^, ^45^, ^46^ Integrating the results of miRNA-seq in exosomes and mRNA sequencing in hearts, a large part of the differentially expressed genes with or without MSC-derived exosome was found to be potentially regulated by major miRNAs contained in hMSC-derived exosomes. These findings are in agreement with the current concept that exosomes transfer miRNAs to regulate host cell gene expression,^25^^,^^26^ but they do not exclude the importance of other bioactive components. We did not evaluate the roles of other extracellular vesicles like membrane vesicles. Although few changes were observed in the numbers of such larger vesicles (Figures 1A, 2C, and S5), our study does not exclude the possibility that such particles play some role.

A growing number of clinical studies have demonstrated the usefulness of stem/progenitor cell transplantation for various diseases.^19^, ^20^, ^21^, ^22^, ^23^ There are still many challenges facing the use of this emerging cell therapy.^53^ In our study, we used systemic injection of hMSCs and developed a new method using gLuc-fusion MFG-E8 to monitor the amount of exosome produced by the injected MSCs. The activities of gLuc-MFG-E8 correlated with the number of exosomes *in vitro* and gradually decreased during the 3 days after injection *in vivo* (Figures 2F and 4B). These results are in sharp contrast to the very fast disappearance of exosomes from the circulation within 2 h by macrophage-mediated phagocytosis in the liver when isolated exosomes were directly injected i.v.^39^^,^^40^ These results suggest that MSC therapy may have a different profile than cell-free exosome injection, at least in maintaining high concentrations of exosomes in the blood circulation.

Our study did not completely rule out the possibility that hMSCs might exert their effect independent of exosomes. However, this study strongly suggested that i.v. administrated hMSCs exerted their effects, at least partly, through exosome secretion in our TAC model. Similar to findings in previous reports, we found that injected hMSCs mainly localized in the lung, not in the heart (Figure 2B). Transplantation of Alix-deficient hMSCs with significantly less exosome production did not improve cardiac function (Figures 7G and 7H), although these Alix-deficient hMSCs exerted profiles of cytokine production and differentiation activities similar to those of the control hMSCs (Figures S7 and S8).

We performed a set of experiments using TAC-induced hypertrophy as a heart failure model and demonstrated that hMSC injections from an early time point prevented cardiac dysfunction. There will be also a possibility that hMSCs exert curative and/or regenerative effects on the heart after the development of heart failure, which remains to be determined. Also, the TAC model does not completely reflect the condition of clinical heart failure. Further studies in different heart failure models should be important. The beneficial effects of hMSC injections for mortality and cardiovascular remodelings in severe conditions, such as end-stage heart failure and myocardial infarction, would be worth testing in the future.

We have established in the present study the importance of circulating adiponectin and exosomes on the cardioprotective properties of MSC therapy in a heart failure mouse model, in which progressive deterioration through loss of adiponectin or T-cadherin in mice was reported.^13^ Adiponectin is a well-known pleiotropic humoral factor with anti-apoptotic, -fibrotic, and -diabetic effects.^2^ Our RNA-sequencing (RNA-seq) analysis identified differential expression of genes associated with transforming growth factor β (TGF-β) and apoptotic signaling following injection of hMSCs, i.e., a gain of MSCs (Figures 8C and 8D), well overlapping with known functional pathways of adiponectin. MSC-like cells reside in virtually all tissues and are thought to maintain organ homeostasis.^17^ Analyses on the expression of T-cadherin in multiple single-cell RNA sequence databases in a variety of tissues suggested that tissue-resident MSC-like cells express T-cadherin (Figure S9). The t-distributed stochastic neighbor embedding (tSNE) cluster positive for PDGFRα (Pdgfra), a marker of MSC-like cells or mesenchymal progenitors,^54^^,^^55^ and Meflin (Islr), which is specifically expressed in mesenchymal progenitors,^56^^,^^57^ contains cells expressing T-cadherin (Cdh13) in different tissues (Figure S9). Enhancement of exosome production from such tissue-resident MSC-like cells may account for at least a part of adiponectin’s pleiotropic organ protections and requires future studies.

## Materials and Methods

### Cell Transplantation

hMSCs were freshly prepared before each experiment. The cells—prepared at three doses: low (0.5 × 10^5^ cells), mid (1.67 × 10^5^ cells), and high (5.0 × 10^5^ cells)—were mixed with saline at one-third dilution and injected using a 27G needle inserted through the tail vein at 2- to 3-day intervals within a period of 2 weeks. In each session, the cells were injected slowly over a period of at least 30 s. For PKH-labeled hMSC transplantation experiments, hMSCs were labeled with a PKH26 red fluorescent dye (Sigma) according to the protocol supplied by the manufacturer. For RNAi experiments, hMSCs were transfected with Silencer Select siRNA (Ambion) using Lipofectamine RNAiMAX Reagent (Life Technologies) according to the protocol supplied by the manufacturer, followed by injection of the cells the next day. For adenovirus-infected cell injection, hMSCs were infected with Gaussia luciferase-fusion MFG-E8 adenovirus, and the infected cells were injected via the tail vein on the next day. The injected cells were used within five passages in all experiments.

### Adiponectin Purification

HMW-APN purification was performed as reported previously.^11^ Briefly, serum samples were obtained from WT mice at 4 days after infection with adenovirus adiponectin and applied onto T-cadherin-Fc conjugated with Protein G Sepharose (GE Healthcare). Adiponectin was eluted with 5 mM EDTA.

### Exosome Isolation

Exosomes were isolated from the cell culture supernatant as described previously.^33^ Briefly, hMSCs were cultured with a xenofree hMSC culture medium with or without HMW-APN for 48 h. Then, the conditioned medium was collected and centrifuged at 800 × *g* for 10 min to deplete floating cells and at 10,000 × *g* for 30 min to remove cell debris. The plasma sample was mixed with thrombin (500 U/mL) for 10 min to remove fibrin, followed by centrifugation at 12,000 × *g* for 20 min. For exosome isolation, the supernatant, plasma, and serum were ultracentrifuged at an average of 110,000 × *g* for 2 h, followed by a washing step of the exosome pellet with Dulbecco’s phosphate-buffered saline with calcium and magnesium [PBS (+)] at an average of 110,000 × *g* for 2 h (TLA100.1 rotor, Beckman Coulter). The exosome pellets were solubilized directly in Laemmli sample buffer. Essentially, none of the mouse serum treatments, overexpression, or RNAi treatment significantly affected cell viability. Concentration and size distribution of exosomes were analyzed by NTA (NanoSight LM10 System, Quantum Design). Serum exosomes were purified by a phosphatidylserine affinity magnetic resin, MagCapture Exosome Isolation Kit PS (Fujifilm Wako Pure Chemical). The recovery rate of exosomes was estimated as 16% by spiking the known amounts of gLuc-fusion MFG-E8-labeled exosomes into mouse serum and purifying by the affinity resin as described earlier.

### Animal Procedures

C57B6/J male mice were purchased from CLEA Japan. AKO mice intensively backcrossed to C57BL/6J background were used.^33^ Pioglitazone (30 mg/kg twice a day [BID], Takeda Pharmaceutical) was administered orally for 15 days. Mice were housed in cages in a room set at 22°C under a 12-h:12-h light:dark cycle (lights off from 8:00 a.m. to 8:00 p.m.). Animals were randomly allocated after sham or TAC operation in all experiments. Data were analyzed in a blinded fashion.

### Cell Culture

The hMSCs were obtained from Lonza and maintained in a xenofree hMSC culture medium (ROHTO Pharmaceutical). For RNAi experiments, hMSCs were transfected with Silencer Select siRNA (Ambion) by using Lipofectamine RNAiMAX reagent (Life Technologies) according to the protocol supplied by the manufacturer. Incubation with adiponectin-containing media started 36 h after transfection. The multilineage differentiation of hMSCs to adipocytes, osteoblasts, and chondrocytes was tested by using Mesenchymal Stem Cell-Adipogenic Differentiation Medium 2, -Osteogenic Differentiation Medium, and -Chondrogenic Differentiation Medium, respectively (PromoCell), according to the protocol supplied by the manufacturer. After the incubation with differentiation medium, staining with Oil Red O, Alizarin Red S, and Alcian Blue was performed to detect the adipocytes, osteoblasts, and chondrocytes, respectively.

### Antibodies

The following primary antibodies were used: goat polyclonal anti-adiponectin (AF1119, R&D Systems); goat polyclonal anti-T-cadherin (AF3264, R&D Systems); rabbit monoclonal anti-α-tubulin (11H10, Cell Signaling Technology); sheep polyclonal anti-human MFG-E8 (AF2767, R&D Systems); mouse monoclonal anti-human CD63 (H5C6, BD Biosciences); rabbit monoclonal anti-Tsg101 (ab125011, R&D Systems); rabbit polyclonal anti-syntenin (ab19903, Abcam); and mouse monoclonal anti-ALIX (3A9, Santa Cruz Biotechnology). The following secondary antibodies were used: horseradish-peroxidase-conjugated (HRP-conjugated) rabbit anti-sheep immunoglobulin G (IgG) (Invitrogen); HRP-conjugated donkey anti-goat IgG (R&D systems); and HRP-conjugated sheep anti-mouse IgG antibodies and donkey anti-rabbit IgG antibody (GE Healthcare).

### Western Blotting

WCLs were loaded onto 4%–20% gradient SDS-PAGE gels (Bio-Rad) and transferred onto nitrocellulose membranes. The membranes were blocked with Block-One blocking reagent (Nakarai Tesque) and then incubated with primary antibodies using Can Get Signal Solution 1 (TOYOBO) overnight at 4°C, followed by incubation with secondary antibodies conjugated with HRP using Can Get Signal Solution 2 (TOYOBO) for 60 min at room temperature. Chemiluminescence signals developed with Chemi-Lumi One Super (Nakarai Tesque) were visualized by ChemiDoc Touch and quantitated using Image Lab software (Bio-Rad).

### Quantitative Real-Time PCR

Total RNA was isolated from mouse tissues by using RNA STAT-60T (Tel-Test, Friendswood, TX, USA) according to the protocol supplied by the manufacturer. First-strand cDNA was synthesized using ReverTra Ace qPCR RT Master Mix (TOYOBO). Quantitative real-time PCR amplification was conducted with QuantStudio7 (Applied Biosystems) using Power SYBR Green PCR Master Mix (Applied Biosystems) according to the protocol recommended by the manufacturer. The sequences of primers used for quantitative real-time PCR were as follows: mouse Rplp0 (36B4): forward (Fw), 5′-GGCCAATAAGGTGCCAGCT-3′, and reverse (Rv), 5′- TGATCAGCCCGAAGGAGAAG-3′; Nppa (ANP): Fw, 5′-GCTTCCAGGCCATATTGGAG-3′, and Rv, 5′-GGGGGCATGACCTCATCTT-3′; Nppb (BNP): Fw, 5′-GAGGTCACTCCTATCCTCTGG-3′, and Rv, 5′-GCCATTTCCTCCGACTTTTCTC-3′; human RPLP0 (36B4): Fw, 5’-GGCGACCTGGAAGTCCAACT-3’, and Rv, 5’-CCATCAGCACCACAGCCTTC-3’; human CDH13 (T-cadherin): Fw, 5’-AGTGTTCCATATCAATCAGCCAG-3’; human RAB27A: Fw, 5’-ACAACAGTGGGCATTGATTTCA-3’, and Rv, 5’-AAGCTACGAAACCTCTCCTGC-3’; human PDCD6IP (Alix): Fw, 5’-ATCGCTGCTAAACATTACCAGTT-3’, and Rv, 5’-AGGGTCCCAACAGTATCTGGA-3’.

### TAC Operation

The TAC operation was performed using the minimally aortic transverse banding method described in detail by Martin et al.^58^ In brief, C57BL/6 male mice (8–9 weeks old, 23–27 g) were anesthetized with a mixture of pentobarbital sodium (50 mg/kg intraperitoneally [i.p.]) and ketamine (25 mg/kg i.p.), as described previously.^16^ The thymus was gently put away to expose the aortic arch. After isolation of the transverse aorta, it was constricted by a 7-0 silk suture ligature fastened stiffly to a 27G needle to yield a constriction of 0.4 mm in diameter. Sham-operated mice underwent a similar surgical procedure, including the exposure of the transverse aorta but without the constriction. The chest was closed with a 5-0 silk suture, and mice were allowed to recover. The procedure was performed under a surgical microscope and was completed in 10 min.

### Echocardiography

Transthoracic echocardiography was performed as described in detail previously.^59^ Briefly, it was performed in each mouse using the LOGIQe ultrasound system with a 4.0- to 10.0-MHz linear probe (i12L-RS) (GE Healthcare). The mouse was first anesthetized with isoflurane and laid on a heating pad to maintain body temperature at 35–37°C. After obtaining the long-axis two-dimensional image of the left ventricle (LV), a two-dimensional guided M-mode trace crossing the septal and posterior walls was recorded. The following parameters were measured on the M-mode tracings: interventricular septal thickness, LV posterior wall thickness, LV end-diastolic diameter (LVDd), LV end-systolic diameter (LVDs), and LV FS [LV FS = (LVDd − LVDs)/LVDd × 100].

### Measurement of Gaussia Luciferase Activity

The exosome fraction was prepared as described earlier in the Exosome Isolation section. The Gaussia luciferase activity in the exosome was measured by using the Gaussia Luciferase Flash Assay Kit (Thermo Fisher Scientific) according to the protocol supplied by the manufacturer.

### RNA-Seq

miRNA-seq was performed at the NGS core facility of the Genome Information Research Center at the Research Institute for Microbial Diseases of Osaka University, Osaka, Japan. Exosomal RNAs derived from hMSCs were isolated using a miRNeasy Mini Kit (QIAGEN). Small RNA libraries were constructed according to the instructions provided by the manufacturer using the NEBNext Small RNA Library Prep Set for Illumina (New England Biolabs) and sequenced by the HiSeq 2500 platform (Illumina) in 75-bp single-end reads. The miRNA-seq analysis was conducted using StrandNGS v.3.0 software (Strand Life Sciences) according to the small RNA alignment and small RNA analysis pipeline using the default parameters. Before analysis of the small RNA-seq data, reads were trimmed of the adaptor sequences and mapped to the human hg19 reference genome. The relative small RNA expression levels were calculated using the DESeq algorithm. The RNA-seq library was prepared using a TruSeq Stranded mRNA sample prep kit (Illumina, San Diego, CA, USA) according to the instructions supplied by the manufacturer. Sequencing was performed on an Illumina HiSeq 2500 platform in a 75-base single-end mode. Illumina Casava v.1.8.2 software was used for base calling. The sequenced reads were mapped to the human reference genome sequences (hg19) using TopHat v.2.0.13 in combination with Bowtie2 v.2.2.3 and SAMtools v.0.1.19. The FPKMs were calculated using Cuffnorm v.2.2.1.

### Cytokine Array

The conditioned medium was collected and centrifuged at 800 × *g* for 10 min to deplete floating cells, and the pooled sample of four separate cells was analyzed by using the Cytokine Array – Human Cytokine Antibody Array (Membrane, 42 Targets, Abcam) according to the protocol supplied by the manufacturer.

### Measurement of Plasma Adiponectin Levels by ELISA

Blood samples were collected from the respective mice, and plasma adiponectin levels were measured by adiponectin ELISA (Otsuka Pharmaceutical) according to the protocol supplied by the manufacturer.

### Histochemistry

The heart tissue was collected from each mouse, and the LV was embedded in paraffin following 10% formalin fixation. Mouse heart sections (4 μm thick) were prepared and stained with hematoxylin and eosin (H&E). Other sections were also stained with CFTM594-conjugated WGA (Biotium) to evaluate the myocyte cross-section area (CSA). For the quantification of CSA, the area was detected and analyzed using a BZ-X700 microscope and the built-in software (Keyence). Mouse lung tissue was collected from mice injected with PKH-labeled hMSCs, and the tissue was embedded in Tissue-Tek O.C.T. Compound (Sakura). Tissue sections prepared were 20 μm thick and stained with DAPI.

### Ethical Considerations

The experimental protocol was approved by the Ethics Review Committee for Animal Experimentation of Osaka University School of Medicine. This study also conforms to the Guide for the Care and Use of Laboratory Animals published by the U.S. National Institutes of Health.

### Statistical Analysis

Data were expressed as mean ± SEM. Differences between the experimental groups were assessed by Student’s t test or one-way ANOVA, followed by post hoc Dunnett’s test and Tukey’s test. The p values < 0.05 were considered statistically significant. All analyses were performed with JMP Software v.13.0 (SAS Institute, Cary, NC, USA).

### Data and Code Accessibility

We submitted all raw datasets except RNA-seq data to DRYAD (https://doi.org/10.5061/dryad.t76hdr7xq). RNA-seq datasets were deposited to the DNA Data Bank of Japan as DDBJ: DRA009757 (in vitro exosome miRNA-seq) and DRA009758 (*in vivo* mouse heart RNA-seq).

## Author Contributions

Y.N. and S.K. designed the research protocol; performed the biochemical, cellular, and *in vivo* experiments; analyzed the data; and co-wrote the manuscript. Y. Tanaka assisted in echo-cardiography. S.F., Y.O., and T.O. assisted in exosome purification and biochemical analysis of exosomes. H.N. cultured hMSCs under the xenofree condition for animal injection. Y.K., Y.T.-S., and Y.F. assisted in animal breeding. Y. Takahashi and Y. Takakura provided gLuc-fusion MFG-E8 cDNA. S.M. and Y.S. provided input on research design and methods for TAC operation. H.N. and N.M. contributed to reviewing and editing the manuscript. S.K. and I.S. directed the research and co-wrote the manuscript, with assistance from all other authors.

## Conflicts of Interest

Osaka University filed a patent claim regarding adiponectin-mediated stimulation of exosome production, the Japanese Patent #6618079 (inventors: S.K., Y.O., N.M., and I.S.). Osaka University and Rohto Pharmaceutical Co. have filed a patent claiming the use of MSCs with pioglitazone for heart failure treatments (the Japan Patent Application #2019-234288, investors: S.K., I.S., N.M., and H.N.).

## Supplemental Information

Document S1. Tables S1–S5 and Figures S1–S9

Document S2. Article plus Supplemental Information
