# Supplementary material for: Adiponectin Stimulates Exosome Release to Enhance Mesenchymal Stem-Cell-Driven Therapy of Heart Failure in Mice
Source: Mol Ther. 2020 Jul 10;28(10):2203–19. doi: 10.1016/j.ymthe.2020.06.026 (PMC7351027; doi:10.1016/j.ymthe.2020.06.026)
Supplement: Document S1. Tables S1–S5 and Figures S1–S9 [file mmc1.pdf]

YMTHE, Volume *28*

Supplemental Information

Adiponectin Stimulates Exosome Release to Enhance Mesenchymal Stem-Cell-Driven Therapy of Heart Failure in Mice

Yuto Nakamura, Shunbun Kita, Yoshimitsu Tanaka, Shiro Fukuda, Yoshinari Obata, Tomonori Okita, Hiroyuki Nishida, Yuki Takahashi, Yusuke Kawachi, Yuri Tsugawa-Shimizu, Yuya Fujishima, Hitoshi Nishizawa, Yoshinobu Takakura, Shigeru Miyagawa, Yoshiki Sawa, Norikazu Maeda, and Iichiro Shimomura

	WT			AKO	
		TAC		TAC	
	Sham	Cont	hMSC	Cont	hMSC
N=	3	5	6	6	6
EF, %	87.30±0.85	70.66±1.34	87.04±2.33	71.74±1.68	73.23±1.36
FS, %	51.59±1.12	34.54±1.05	51.26±2.88	35.33±1.29	36.07±1.03
IVSd, mm	1.22±0.05	1.52±0.14	1.28±0.08	1.71±0.14	1.53±0.05
IVSs, mm	1.87±0.03	1.74±0.13	1.97±0.10	2.15±0.13	1.89±0.05
LVIDd, mm	3.59±0.22	3.24±0.17	3.21±0.21	2.86±0.10	2.85±0.19
LVIDs, mm	1.74±0.14	2.12±0.13	1.59±0.19	1.85±0.07	1.83±0.14
LVPWd, mm	1.14±0.14	1.55±0.20	1.41±0.07	1.93±0.10	1.71±0.08
LVPWs, mm	1.71±0.09	2.09±0.17	1.92±0.14	2.25±0.09	2.03±0.09
SV, μL	45.51±5.84	27.72±3.42	34.55±4.43	23.48±2.13	21.23±4.99
CO, mL/min	25.06±3.93	13.29±1.35	18.53±2.72	12.58±1.82	11.94±2.85
HR, beats/min	549±30	485±19	532±24	531±29	548±16

Table S1. Echocardiographic evaluation of the effects of human adipose-derived mesenchymal stem cells on cardiac function in adiponectin knockout mice.
Data are mean±SEM.
Human adipose tissue-derived MSCs (hMSCs) were injected intravenously into the transverse aortic constriction (TAC) model in AKO and WT mice. hMSCs were injected at concentration of $5.0x10^5$ cells/body via the tail vein. Injections were repeated six times at 2-3 day intervals within a period of 2 weeks. Echocardiography was performed at day14.
EF: Ejection Fraction. FS: Fractional Shortening. IVSd: interventricular septum thickness in diastole. IVSs: interventricular septum thickness in systole. LVIDd: left ventricular internal dimension in diastole. LVIDs: left ventricular internal dimension in systole. LVPWd: left ventricular posterior wall thickness in diastole. LVPWs: left ventricular posterior wall thickness in systole. SV: Stroke Volume. CO: Cardiac Output. HR: Heart Rate.

	WT					AKO	
		TAC				TAC	
		Pio (-)		Pio (+)		Pio (+)	
	Sham	Cont	hMSC	Cont	hMSC	Cont	hMSC
N=	3	4	6	4	9	4	7
EF, %	88.14±1.47	74.38±1.05	81.09±0.69	74.65±2.10	85.77±0.80	71.80±0.79	72.69±1.10
FS, %	52.16±1.99	37.52±0.91	43.59±0.69	37.79±1.75	48.93±0.99	35.31±0.63	36.12±0.81
IVSd, mm	0.90±0.04	1.51±0.05	1.38±0.05	1.45±0.01	1.20±0.06	1.52±0.02	1.50±0.05
IVSs, mm	1.87±0.03	1.48±0.06	2.02±0.03	1.87±0.06	1.89±0.08	1.94±0.09	1.87±0.07
LVIDd, mm	3.62±0.28	3.38±0.06	3.02±0.12	3.15±0.09	3.09±0.09	3.00±0.18	2.80±0.21
LVIDs, mm	1.73±0.14	2.11±0.04	1.70±0.08	1.96±0.09	1.58±0.06	1.94±0.12	1.80±0.15
LVPWd, mm	0.94±0.07	1.52±0.03	1.41±0.05	1.53±0.07	1.32±0.07	1.49±0.02	1.49±0.02
LVPWs, mm	1.66±0.14	1.98±0.09	1.91±0.07	1.87±0.11	1.89±0.06	1.86±0.06	2.00±0.06
SV, μL	47.17±8.97	32.22±1.50	27.38±2.52	27.36±1.90	30.94±2.08	23.65±3.08	20.87±3.34
CO, mL/min	27.44±5.61	17.69±0.60	15.94±1.43	14.05±1.04	18.37±1.33	12.70±1.73	11.47±1.64
HR, beats/min	579±8	550±14	584±19	518±43	596±21	539±27	564±21

Table S2. Echocardiographic evaluation of the effects of human adipose-derived mesenchymal stem cells on cardiac function in pioglitazone-treated mice.
Data are mean±SEM.
Wild-type (WT) and Adiponectin knockout (AKO) mice of the transverse aortic constriction (TAC) model received intravenous injections of human adipose tissue-derived MSCs (hMSCs) and oral pioglitazone (Pio; 30 mg/mL, p.o, BID). hMSCs were injected at a concentration of $1.67x10^5$ cells/body via the tail vein. The inject was repeated six times at 2-3 day intervals within a period of 2 weeks. Echocardiography was performed at day14.
Abbreviations as in Table S1.

		TAC		
			hMSC	
	Sham	Cont	siCont	siTcad
N=	5	7	7	6
EF, %	87.76±1.56	74.13±1.02	87.25±1.02	80.57±0.49
FS, %	51.88±2.26	37.25±0.80	50.88±1.36	42.90±0.50
IVSd, mm	0.95±0.07	1.53±0.05	1.23±0.10	1.43±0.07
IVSs, mm	1.61±0.12	2.00±0.07	1.84±0.14	1.87±0.08
LVIDd, mm	3.58±0.10	3.06±0.18	3.07±0.20	2.52±0.10
LVIDs, mm	1.73±0.11	1.92±0.13	1.52±0.13	1.44±0.05
LVPWd, mm	0.86±0.05	1.55±0.12	1.33±0.05	1.57±0.06
LVPWs, mm	1.46±0.03	1.82±0.16	1.93±0.08	1.95±0.11
SV, μL	44.96±2.53	25.70±2.94	31.61±4.92	17.55±1.59
CO, mL/min	24.21±0.91	12.75±1.31	16.72±1.99	9.19±1.08
HR, beats/min	543±29	502±20	547±28	517±27

Table S3. Echocardiographic evaluation of T-cadherin knockdown on the effect of human adipose-derived mesenchymal stem cells on cardiac function in wild-type mice.
Data are mean±SEM.
Human adipose tissue-derived MSCs (hMSCs) were transfected with control or T-cad RNAi and injected at a concentration of $5.0x10^5$ cells/body via the tail vein the day after transfection. The injection was repeated six times at 2-3 day intervals within a period of 2 weeks. Echocardiography was performed at day14.
Abbreviations as in Table S1.

		TAC		
			hMSC	
	Sham	Cont	siCont	siAlix
N=	4	6	8	9
EF, %	88.60±1.15	74.50±0.80	86.04±0.95	76.01±1.52
FS, %	52.70±1.71	37.50±0.67	49.26±1.16	38.96±1.31
IVSd, mm	1.14±0.10	1.57±0.06	1.23±0.04	1.64±0.06
IVSs, mm	1.78±0.16	1.88±0.09	1.84±0.10	2.13±0.08
LVIDd, mm	3.19±0.17	2.98±0.19	2.97±0.10	2.91±0.15
LVIDs, mm	1.51±0.08	1.86±0.12	1.50±0.06	1.78±0.09
LVPWd, mm	1.13±0.06	1.60±0.06	1.18±0.04	1.49±0.03
LVPWs, mm	1.69±0.09	2.09±0.10	1.87±0.07	1.92±0.06
SV, μL	35.00±4.44	24.49±3.51	28.23±2.36	23.84±2.81
CO, mL/min	20.41±3.04	13.34±1.94	15.12±1.49	13.04±1.62
HR, beats/min	580±30	545±14	534±21	547±13

Table S4. Echocardiographic evaluation of Alix knockdown on the effects of human adipose-derived mesenchymal stem cells on cardiac function in wild-type mice.
Data are mean±SEM.
Human adipose tissue-derived MSCs (hMSCs) were transfected with control or Alix RNAi and intravenously injected at a concentration of $5.0x10^5$ cells/body via the tail vein the day after transfection. Injections were repeated six times at 2-3 day intervals within a period of 2 weeks.
Echocardiography was performed at day14.
Abbreviations as in Table S1.

Accession	FPKM	Accession	FPKM
let-7 family	17888	hsa-mir-125b-1	379
hsa-mir-21	8472	hsa-mir-370	350
hsa-mir-100	7630	hsa-mir-379	326
hsa-mir-148a	7156	hsa-mir-99b	309
hsa-mir-10	5098	hsa-mir-654	297
hsa-mir-26	5035	hsa-mir-30a	280
hsa-mir-199	3975	hsa-mir-122	263
hsa-mir-99a	2707	hsa-mir-125b-2	253
hsa-mir-222	2371	hsa-mir-134	250
hsa-mir-221	1712	hsa-mir-615	238
hsa-mir-27a	1655	hsa-mir-191	230
hsa-mir-127	1608	hsa-mir-6087	203
hsa-mir-22	1534	hsa-mir-532	190
hsa-mir-381	1479	hsa-mir-196a-2	158
hsa-mir-146b	1478	hsa-mir-503	151
hsa-mir-151a	1141	hsa-mir-193a	149
hsa-mir-409	1099	hsa-mir-23a	149
hsa-mir-7704	1083	hsa-mir-196a-1	144
hsa-mir-423	990	hsa-mir-28	141
hsa-mir-320a	917	hsa-mir-382	138
hsa-mir-128-1	685	hsa-mir-455	132
hsa-mir-493	658	hsa-mir-155	127
hsa-mir-92a-1	615	hsa-mir-148b	123
hsa-mir-29a	595	hsa-mir-4497	123
hsa-mir-92a-2	588	hsa-mir-495	123
hsa-mir-128-2	561	hsa-mir-224	121
hsa-mir-218-1	553	hsa-mir-204	119
hsa-mir-218-2	548	hsa-mir-424	119
hsa-mir-378a	525	hsa-mir-143	118
hsa-mir-24-2	523	hsa-mir-30e	118
hsa-mir-27b	522	hsa-mir-320b-1	115
hsa-mir-24-1	521	hsa-mir-1307	110
hsa-mir-7-1	519	hsa-mir-192	110
hsa-mir-7-2	512	hsa-mir-125a	108
hsa-mir-7-3	512	hsa-mir-320b-2	108
hsa-mir-30d	501	hsa-mir-3195	101
hsa-mir-25	423	hsa-mir-140	100
hsa-mir-543	386		

Table S5. MicroRNAs in exosomes from human adipose tissue-derived mesenchymal stem cells (hMSCs).
hMSCs-derived exosomes were collected from the conditioned medium after 48-hrs cultures. Only exosome-derived miRNAs of more than 100 FPKM are shown.

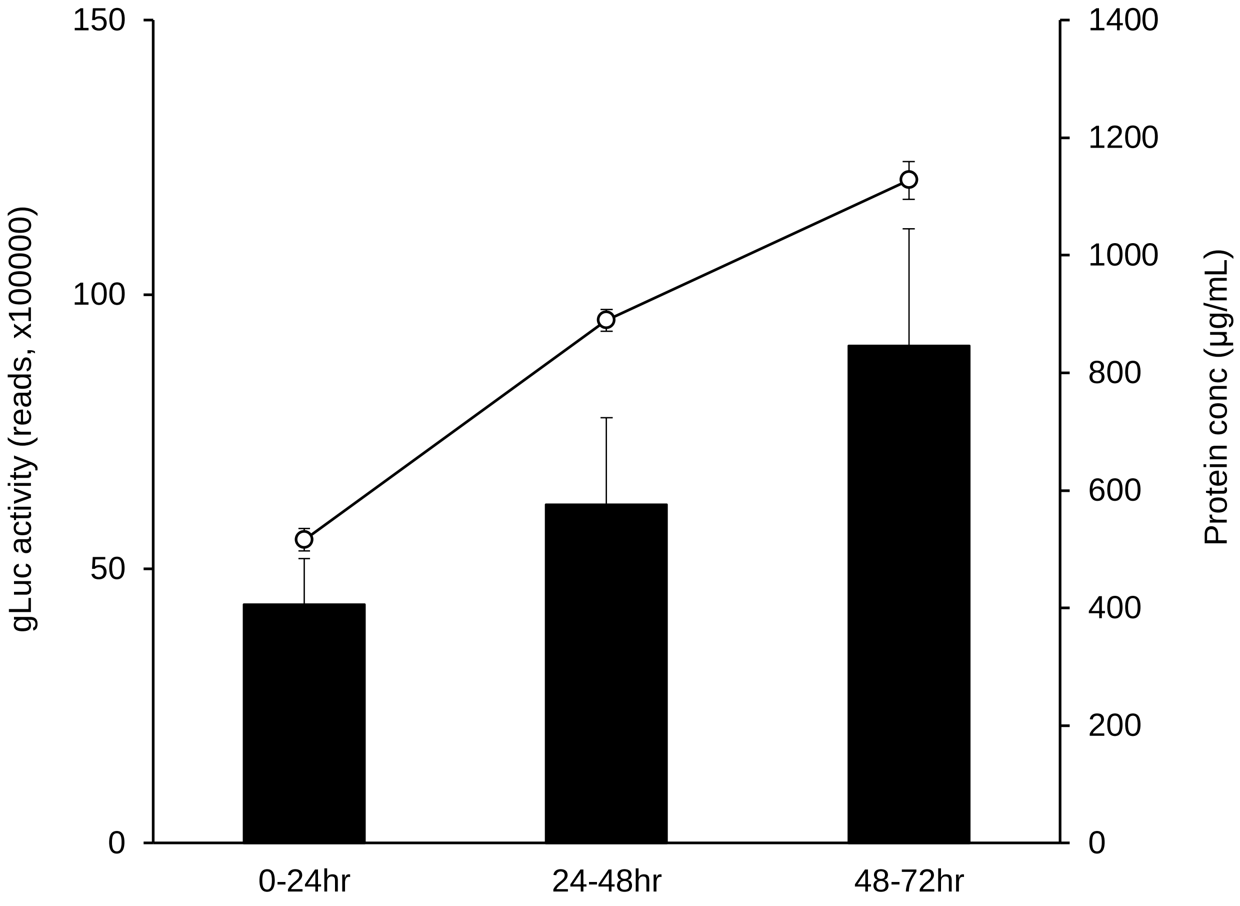

Figure S1. gLuc activity in exosomes from human adipose tissue-derived mesenchymal stem cells infected with gLuc-MFG-E8.
Human adipose tissue-derived mesenchymal stem cells (hMSCs) were transfected with adenovirus *Gaussia* luciferase fused-MFG-E8 (gLuc-MFG-E8). The culture medium was supplied at 24hr-cycles, followed by collections of the conditioned medium. Exosomes in the conditioned media were precipitated by ultracentrifugation as described in the Methods section. *Solid bars:* gLuc activity in exosomes, *line:* protein concentrations in cell lysates at the indicated time points. Data are mean±SEM.

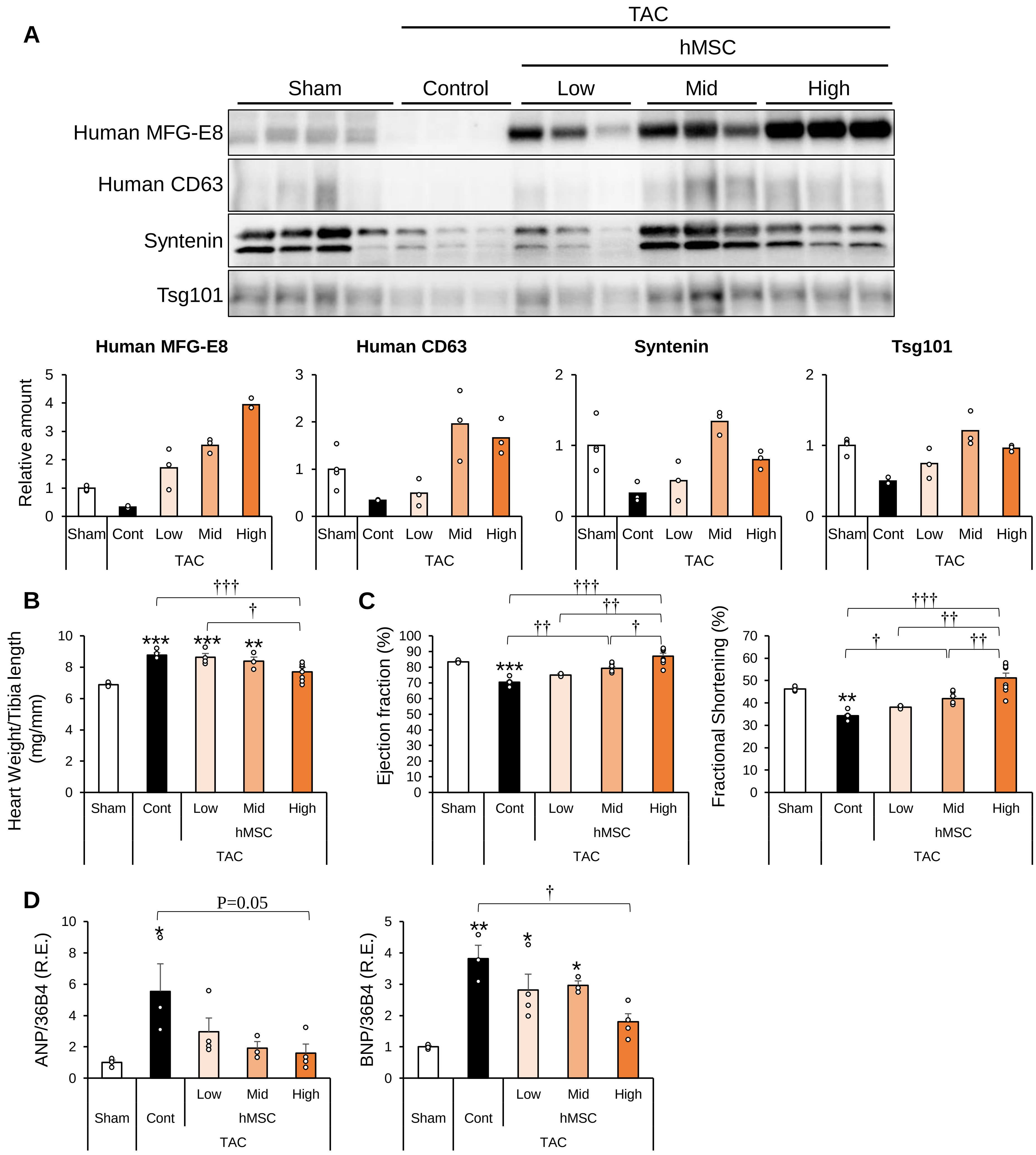

Figure S2. Evaluation of serum exosomes produced by human adipose tissue-stimulated mesenchymal stem cells injected into mice with load-induced heart failure.

Human adipose tissue-derived mesenchymal stem cells (hMSCs) were injected intravenously in load-induced mouse models at a concentration of 0.5 (Low), 1.67 (Mid) and 5.0 (High) x10^5 cells/body via the tail vein. Injections were repeated six times at 2-3 day intervals within a period of 2 weeks. Analysis was performed at day14. **A**, Serum exosomes were subjected to western blot analysis with the indicated antibodies against typical exosome markers (n=3-4). **B**, The ratio of Heart weight/Tibia length (mg/mm, n=3-8). **C**, Echocardiography analysis (n=3-8). **D**, Relative expression (R.E.) of indicated genes (n=3). All genes were normalized by 36B4. Data are mean±SEM. *P<0.05, **P<0.01, ***P<0.001 vs sham. †P<0.05, ††P<0.01, †††P<0.001 between groups by one-way analysis of variance with *post hoc* Tukey's multiple comparisons.

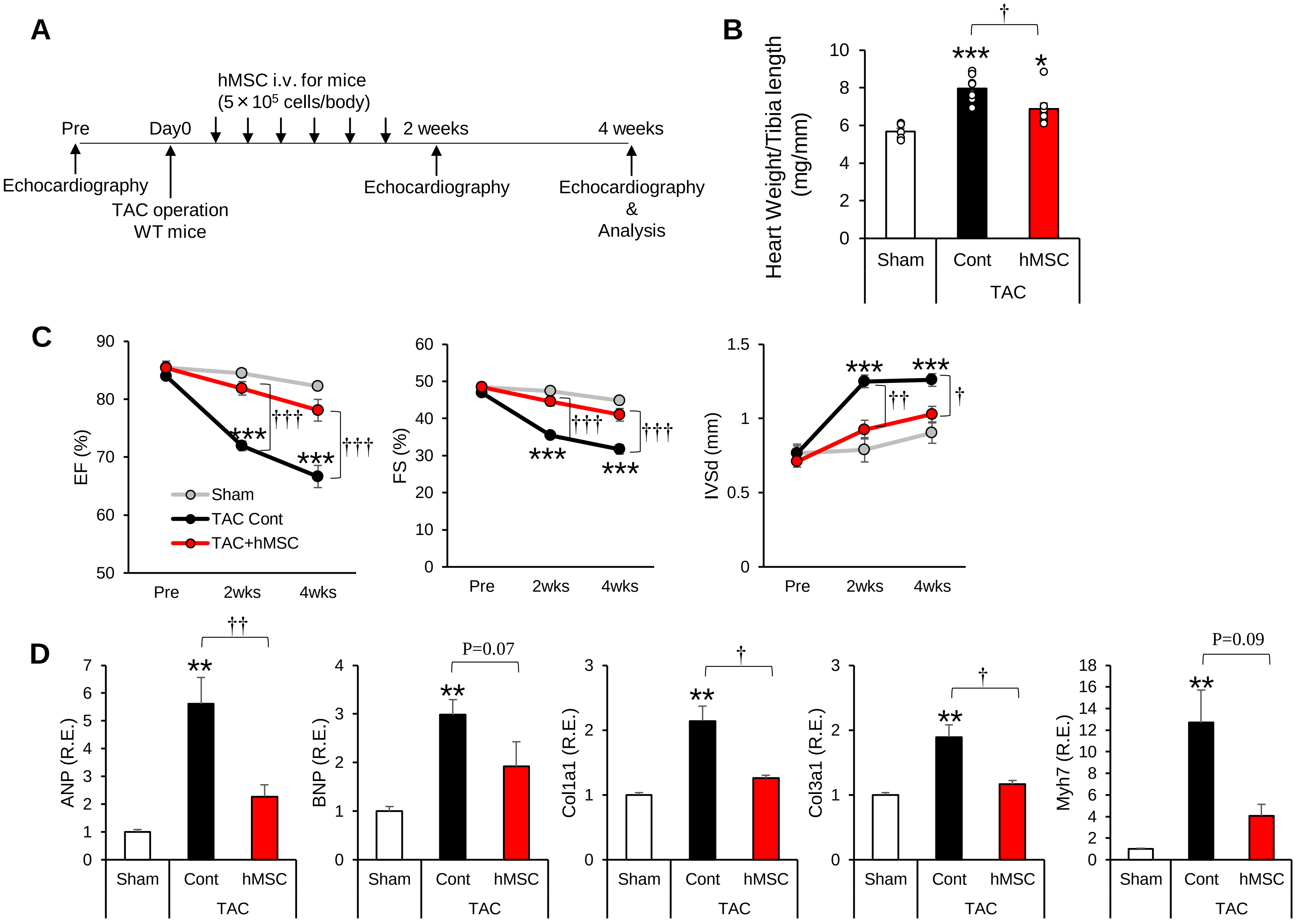

Figure S3. Characteristics of wild-type mice injected human adipose tissue-derived MSCs (hMSCs) were analyzed at 4 weeks after surgery. Intravenous injection of human adipose tissue-derived MSCs (hMSCs) improves cardiac function in mice with pressure overload-induced heart failure. **A**, Experimental design for hMSCs delivery into transverse aortic constriction (TAC) model. hMSCs were injected at concentration of 5.0x10^5 cells/body via the tail vein. The injection was repeated 6 times at 2-3 day intervals within a period of 2 weeks. Echocardiography was performed at 2 and 4 weeks after surgery. **B**, Heart weight per tibia length ratio at 4 weeks after surgery (n=4-8). **C**, Ejection fraction (EF) and fractional shortening (FS) measured at before surgery (Pre) and 2 and 4 weeks after TAC or sham surgery (n=4-8). **D**, Relative expression of heart failure markers of the indicated mice at 4 weeks after surgery (n=4-5). Data are mean±SEM. *P<0.05, **P<0.01, ***P<0.001 vs sham. †P<0.05 , ††P<0.01, †††P<0.001 between groups, by one-way analysis of variance with *post hoc* Tukey's multiple comparisons.

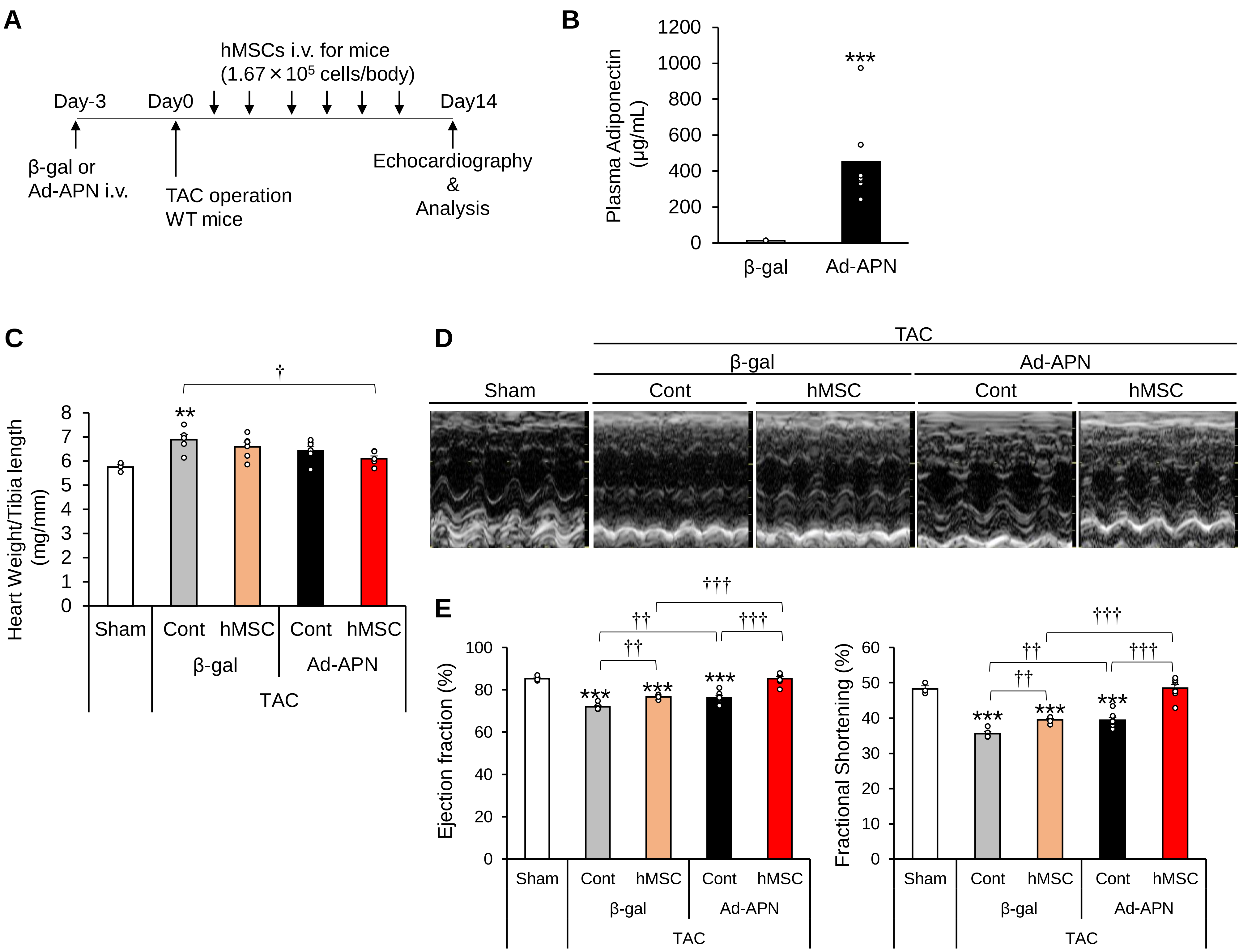

Figure S4. Effects of adiponectin overexpression on the cardioprotective effects of human adipose-derived mesenchymal stem cells (hMSCs). **A**, Experimental design of intravenous injection of hMSCs transfected with adenovirus adiponectin and analysis of their cardioprotective effects in mice of the transverse aortic constriction (TAC) model. hMSCs were injected at a concentration of 1.67×10^5 cells/body via the tail vein. Injection was repeated six times at 2-3 day intervals within a period of 2 weeks. Echocardiography was performed at day14. **B**, Plasma adiponectin level was analyzed by ELISA at Day 0 (n=1). **C**, Heart weight per tibia length ratio (n=3-8). **D**, Representative images of echocardiography. **E**, Ejection fraction and fractional shortening at 2 weeks after TAC or sham surgery (n=3-8). Data are mean±SEM. **P<0.01, ***P<0.001 vs sham. †P<0.05, ††P<0.01, †††P<0.001 between groups by one-way analysis of variance with *post hoc* Tukey's multiple comparisons.

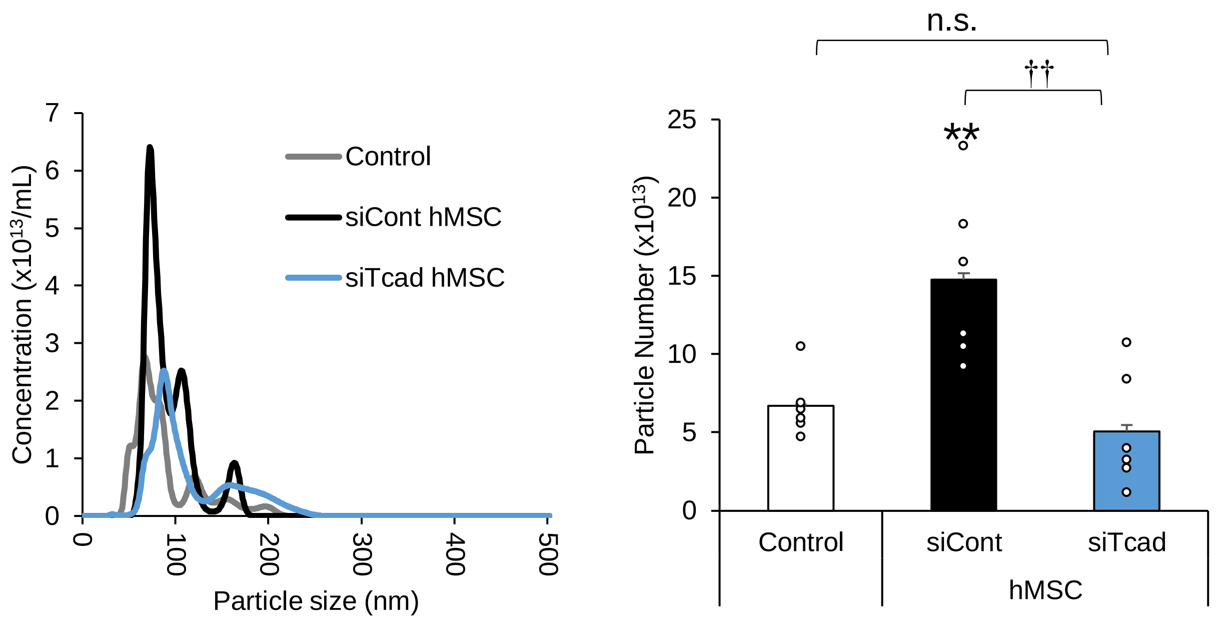

Figure S5. Nanoparticle tracking analysis of serum exosomes from mice injected hMSCs via the tail vein.

Nanoparticle tracking analysis (NTA) was performed using exosome precipitated by serum. The particle size and concentration of serum exosomes were shown (n=6). Control or T-cadherin siRNA transfection was conducted before a day of injection for mice. siRNA transfected-hMSCs were injected via tail vein ($5x10^5$ cells/body) and the serum was collected from each mice at 4hr after injection. Serum exosome was purified by the MagCapture^{TM} Exosome Isolation Kit PS (Wako) and analyzed by NTA. The results of the size and concentration of exosomes in Control and siCont hMSC are the same shown in Figure 2C. Data are mean±SEM. **P<0.01 vs control. ††P<0.01 between groups by one-way analysis of variance with *post hoc* Tukey's multiple comparisons.

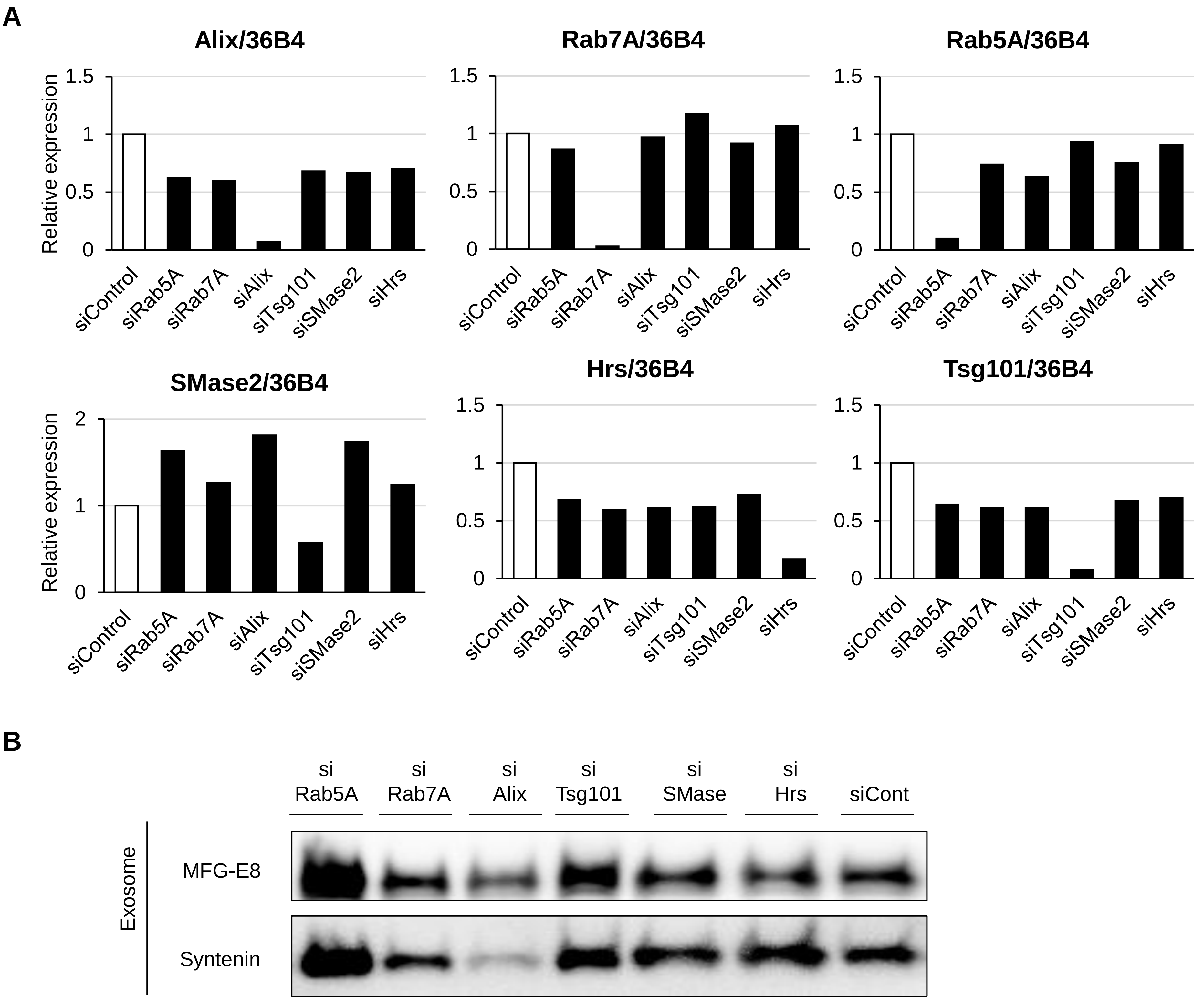

Figure S6. Exosomes from human adipose tissue-derived mesenchymal stem cells.

A, Human adipose tissue-derived MSCs (hMSCs) were transfected with control, Alix, Rab7a, Rab5A, Neutral sphingomyelinases (SMase), or Tsg101 RNAi and subjected to qPCR analysis at 3 days after RNAi transfection (n=1). **B**, Exosomes present in the culture medium at 48hr after transfection were precipitated by ultracentrifugation and subjected to western blot analysis with the indicated antibodies against typical exosome markers (n=1).

A

siC siC+APN siAlix siAlix+APN

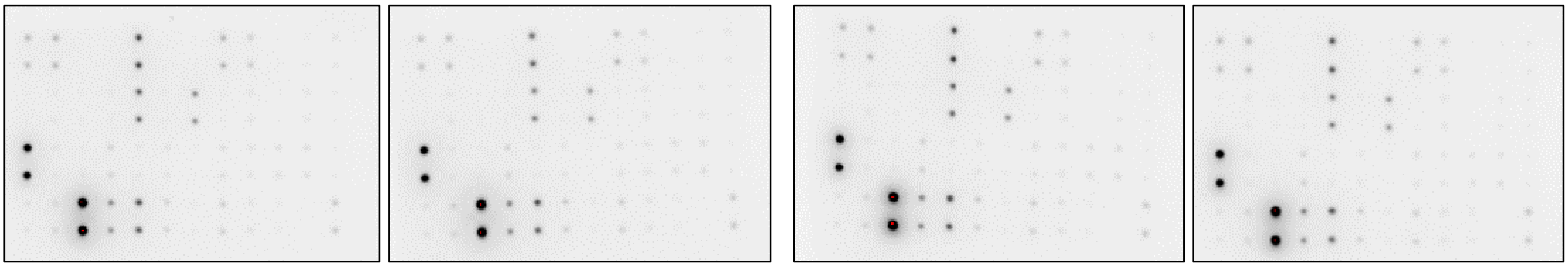

	A	B	C	D	E	F	G	H	I	J	K	L
1	Posi	Posi	Nega	Nega	EMA-78	GCSF	GM-CSF	GRO	GRO-α	I-309	IL-1α	IL-1β
2	Posi	Posi	Nega	Nega	EMA-78	GCSF	GM-CSF	GRO	GRO-α	I-309	IL-1α	IL-1β
3	IL-2	IL-3	IL-4	IL-5	IL-6	IL-7	IL-8	IL-10	IL-12	IL-13	IL-15	IFNγ
4	IL-2	IL-3	IL-4	IL-5	IL-6	IL-7	IL-8	IL-10	IL-12	IL-13	IL-15	IFNγ
5	MCP-1	MCP-2	MCP-3	MCSF	MDC	MIG	MIP-1	RANTES	SCF	SDF-1	TARC	TGFβ1
6	MCP-1	MCP-2	MCP-3	MCSF	MDC	MIG	MIP-1	RANTES	SCF	SDF-1	TARC	TGFβ1
7	TNFα	TNFβ	EGF	IGF-1	Angiogenin	Oncostatin M	Thrombopoietin	VEGF	PDGF BB	Leptin	Nega	Posi
8	TNFα	TNFβ	EGF	IGF-1	Angiogenin	Oncostatin M	Thrombopoietin	VEGF	PDGF BB	Leptin	Nega	Posi

B

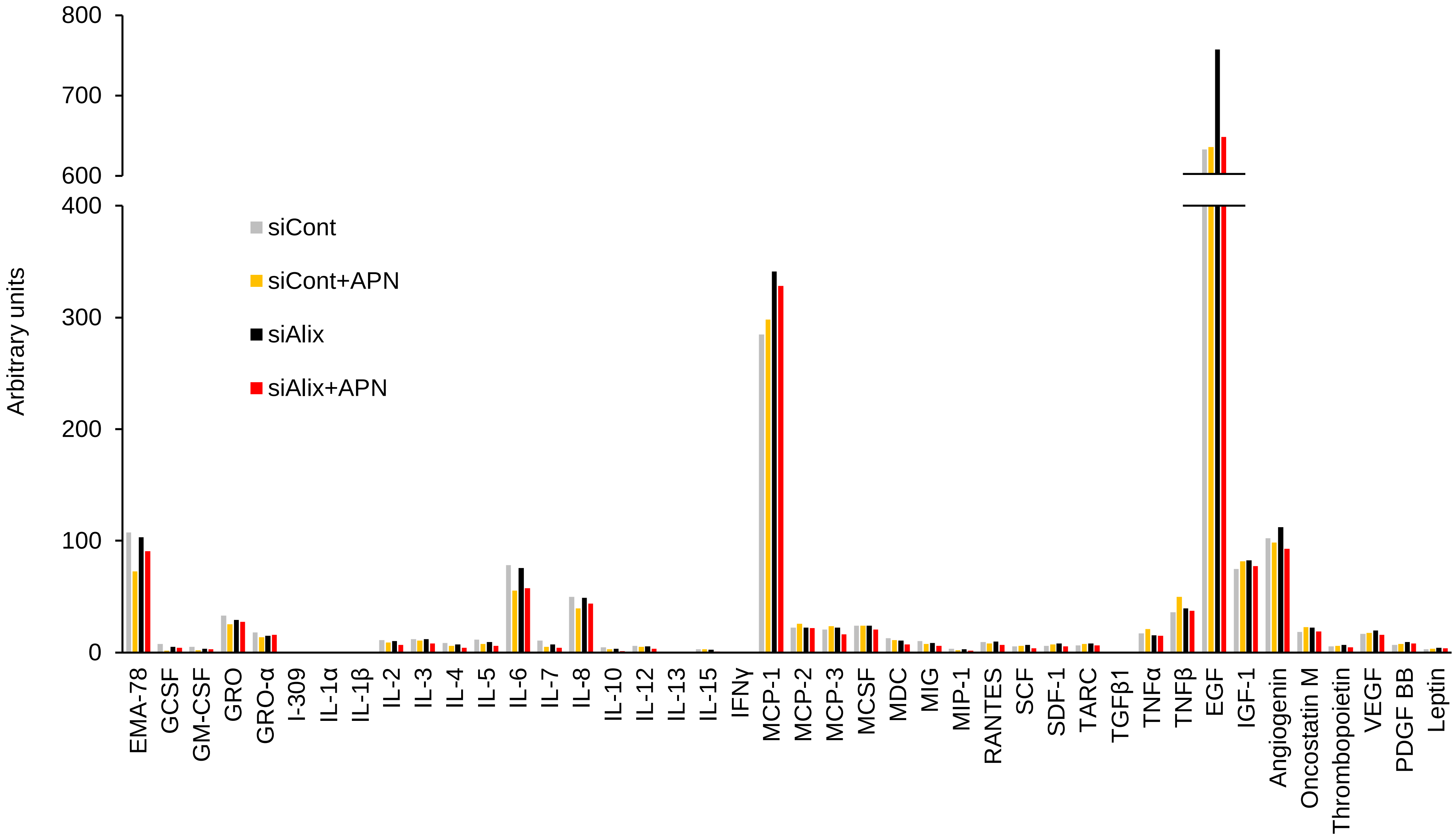

Figure S7. Evaluation of cytokine production by human adipose tissue-derived mesenchymal stem cells.

Human adipose tissue-derived MSCs (hMSCs) were transfected with control or Alix RNAi, with or without high molecular weight adiponectin (20 mg/mL) and subjected to cytokine array analysis at 3 days after RNAi transfection (pooled samples of four separate wells). **A**, Representative blot of cytokine array. **B**, The amounts of the indicated cytokines (arbitrary units).

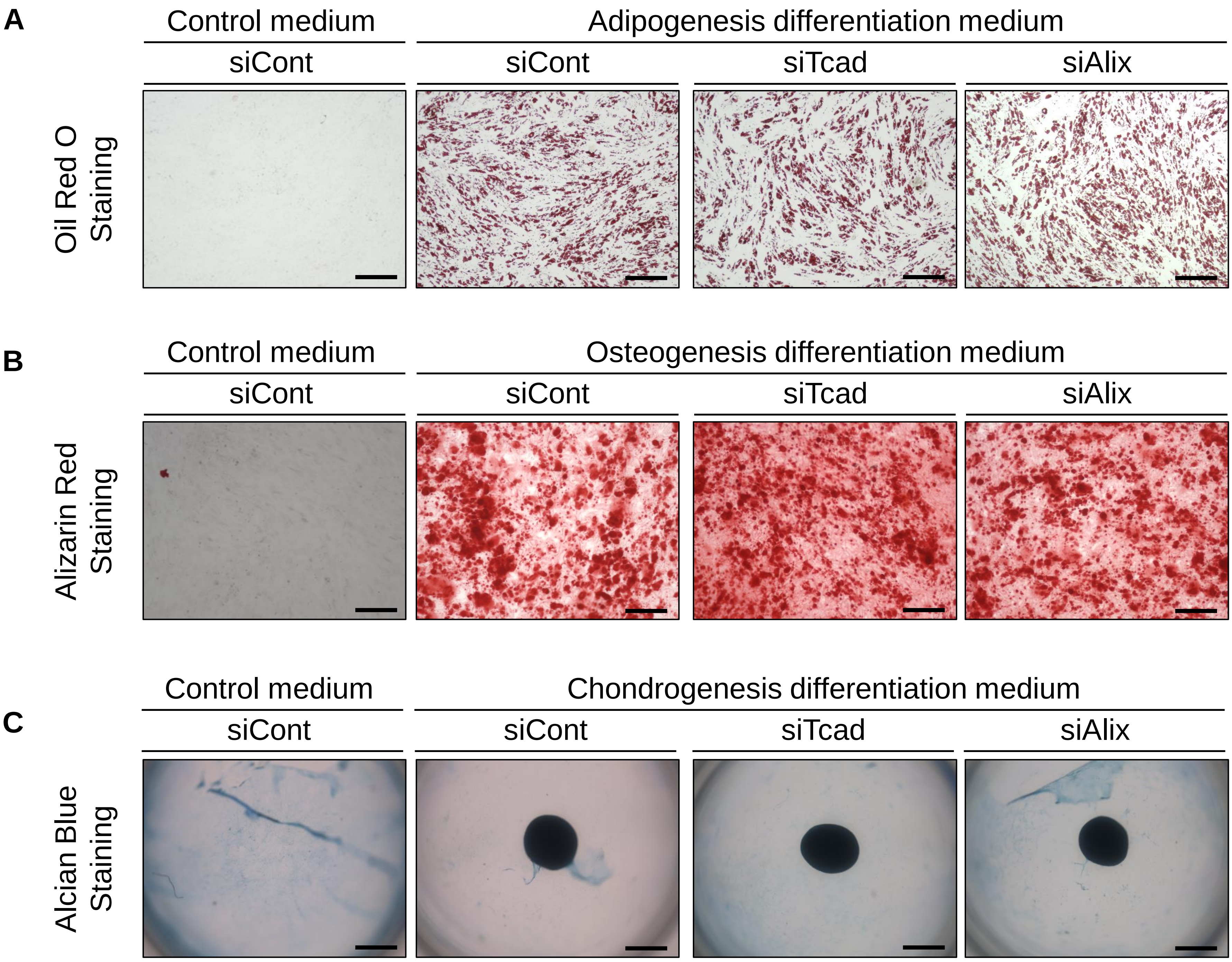

Figure S8. Evaluation of differentiation ability in human adipose tissue-derived mesenchymal stem cells.

Human adipose tissue-derived MSCs (hMSCs) were transfected with control, T-cad or Alix RNAi and subjected to differentiation analysis after RNAi transfection. **A**, Oil Red O staining of hMSCs cultured in adipogenesis differentiation medium for 14 days. Scale bar; 200 µm. **B**, Alizarin Red S staining of hMSCs cultured in osteogenesis differentiation medium for 14 days. Scale bar; 200 µm. **C**, Alcian Blue staining of hMSCs cultured in chondrogenesis differentiation medium for 21 days. Scale Bar; 1 mm.

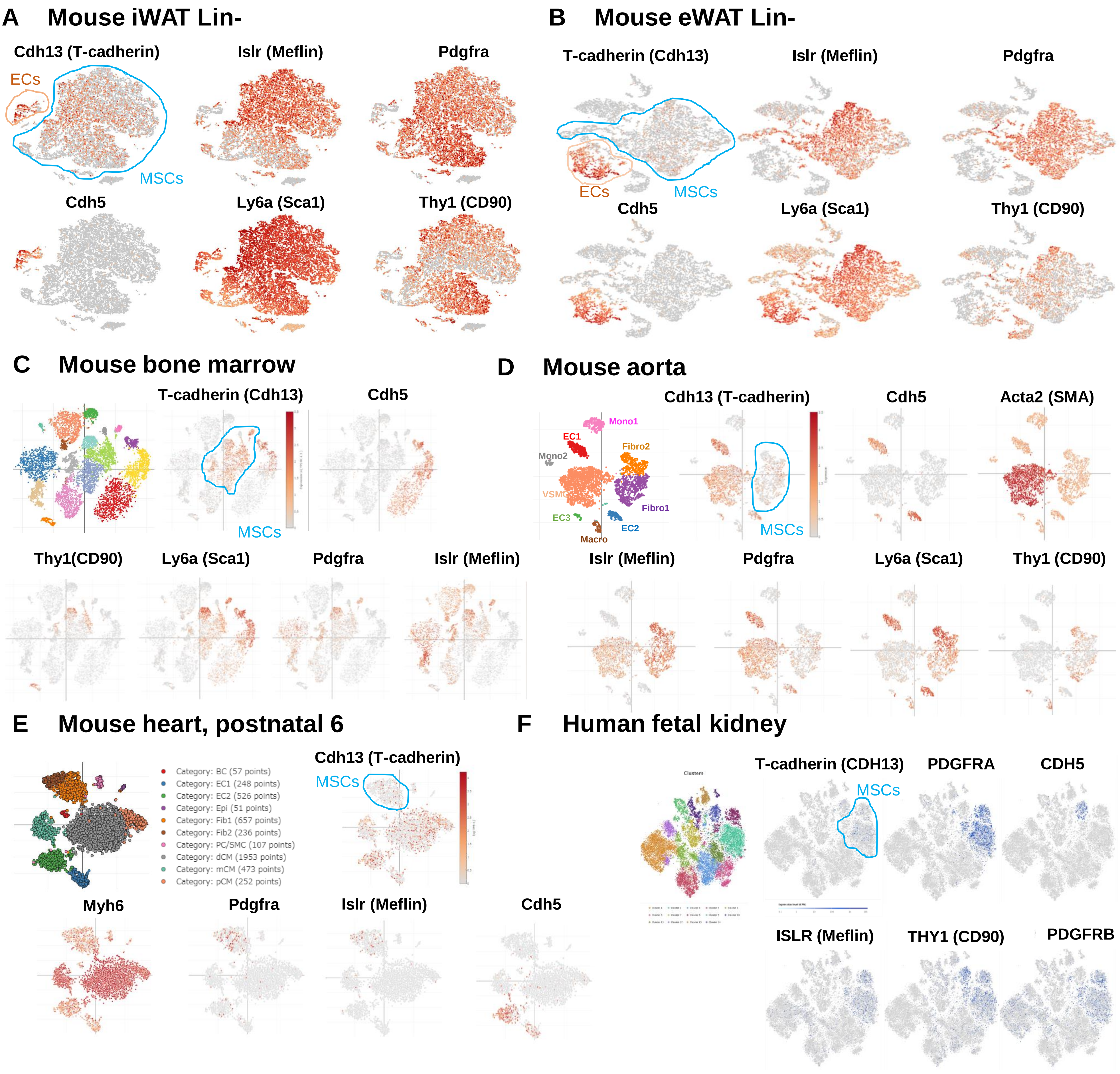

Figure S9. Analyses on the expression of T-cadherin in multiple single-cell RNA sequence databases in a variety of tissues.

T-cadherin (mouse Cdh13, human CDH13) expression in public single cell RNA (scRNA) sequence database. **A-B**, Lineage negative fractions of normal mouse inguinal and epididymal white adipose tissue scRNA-seq libraries (SRP145475) was analyzed as described in original research**. C-E**, tSNE plots of scRNA-seq was obtained from Single Cell Portal (https://singlecell.broadinstitute.org/single_cell). **C**, Normal mouse bone marrow. D, Normal mouse aorta. E, Postnatal p6 mouse heart. F, tSNE plot of scRNA-seq was obtained from Single Cell Expression Atlas (https://www.ebi.ac.uk/gxa/sc/home). Human fetal kidney.
